# Tuning the Ultimate Strain of Single and Double Network Gels Through Reactive Strand Extension

**DOI:** 10.1021/acscentsci.5c00932

**Published:** 2025-08-15

**Authors:** Xujun Zheng, Chun-Yu Chiou, Sunay Dilara Ekim, Tatiana B. Kouznetsova, Jafer Vakil, Yixin Hu, Liel Sapir, Danyang Chen, Zi Wang, Michael Rubinstein, Jian Ping Gong, Nancy R. Sottos, Stephen L. Craig

**Affiliations:** † Center for the Chemistry of Molecularly Optimized Networks, 3065 Duke University, Durham, North Carolina 27708, United States; ‡ Department of Chemistry, 3065 Duke University, Durham, North Carolina 27708, United States; § Department of Materials Science and Engineering, 14589 University of Illinois at Urbana−Champaign, Urbana, Illinois 61801, United States; ∥ Department of Mechanical Engineering and Materials Science, 3065 Duke University, Durham, North Carolina 27708, United States; ⊥ Department of Chemistry and the Institute of Nanotechnology and Advanced Materials, 26731 Bar-Ilan University, Ramat-Gan 52900, Israel; # Departments of Physics and Biomedical Engineering, 3065 Duke University, Durham, North Carolina 27708, United States; ○ Institute for Chemical Reaction Design and Discovery, 12810 Hokkaido University, Sapporo 001-0021, Japan; ◆ Faculty of Advanced Life Science, 12810 Hokkaido University, Sapporo 001-0021, Japan

## Abstract

The stretchability (ability to be elongated) and toughness (capacity to absorb energy before breaking) of polymer network materials, such as elastomers and hydrogels, often determine their utility and lifetime. Direct correlations between the molecular behavior of polymer network components and the physical properties of the network inform the design of materials with enhanced performance, extended lifetime, and minimized waste stream. Here, we report the impact of the fused ring size in bicyclic cyclobutane mechanophores within the strands of polymer network gels. The mechanophores and their polymer strands share the same initial covalent contour length, whereas the capacity for reactive strand extension (RSE) is varied by changing the size of the ring fused to the cyclobutane from 5 to 12 carbon atoms. We observe the first evidence of covalent RSE effects in a single-network gel, and strands with greater RSE lead to gels with greater stretchability and toughness. The same qualitative correlation between molecular and macroscopic extension is also observed in DN hydrogels with mechanophores in the prestretched first network.

## Graphical abstract

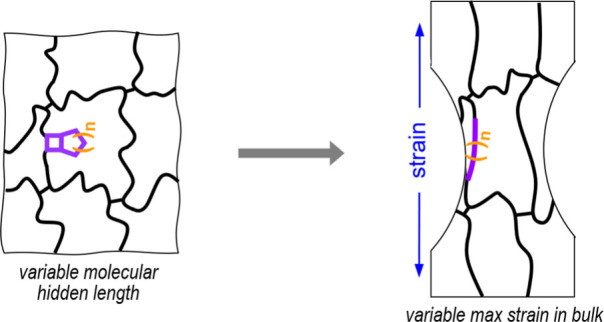

## Introduction

The mechanical fracture of polymeric network materials such as elastomers and hydrogels often defines their utility and determines their lifetime.^1,2^ As a result, approaches to control—and especially to improve—the strains that a given material can attain before breaking are desirable.^3^ To that end, a wide range of toughening strategies have been investigated to great success, including the use of multinetwork architectures,^4,5^ reversible cross-linking,^6,7^ noncovalent domain unfolding,^8^ the addition of fillers,^9^ force-induced recombination,^10^ phase-separated microstructures,^11^ hybrid organic–inorganic composites,^12^ and the incorporation of sacrificial bonds.^13^

In recent years, a complementary approach involving embedded chemical reactivity has emerged. It has been shown that the strategic incorporation of sufficiently reactive mechanophores into polymer networks can provide latent chemical reactivity that does not change low-strain mechanical properties, but that activates when and where needed to counteract processes that lead to material failure. We recently reported double-network (DN) hydrogels comprising strands in the stress-bearing, prestrained network that are capable of reactive strand extension (RSE) via in situ covalent polymer mechanochemistry.^14^ As described in Figure 1a, the RSE motifs lengthen through force-coupled reactions as they reach their nominal breaking point. When the RSE networks are compared to non-RSE controls comprising comonomers that react to break the strand rather than lengthening it, the two networks have indistinguishable low-strain behavior, but the introduction of RSE for scission leads to hydrogels that stretch 40% to 50% further before breaking and exhibit tearing energies that are twice as large. These observations motivate further study of the relationship between the covalent chemical response in polymer strands and the resulting properties in polymer networks.

**Figure 1:**
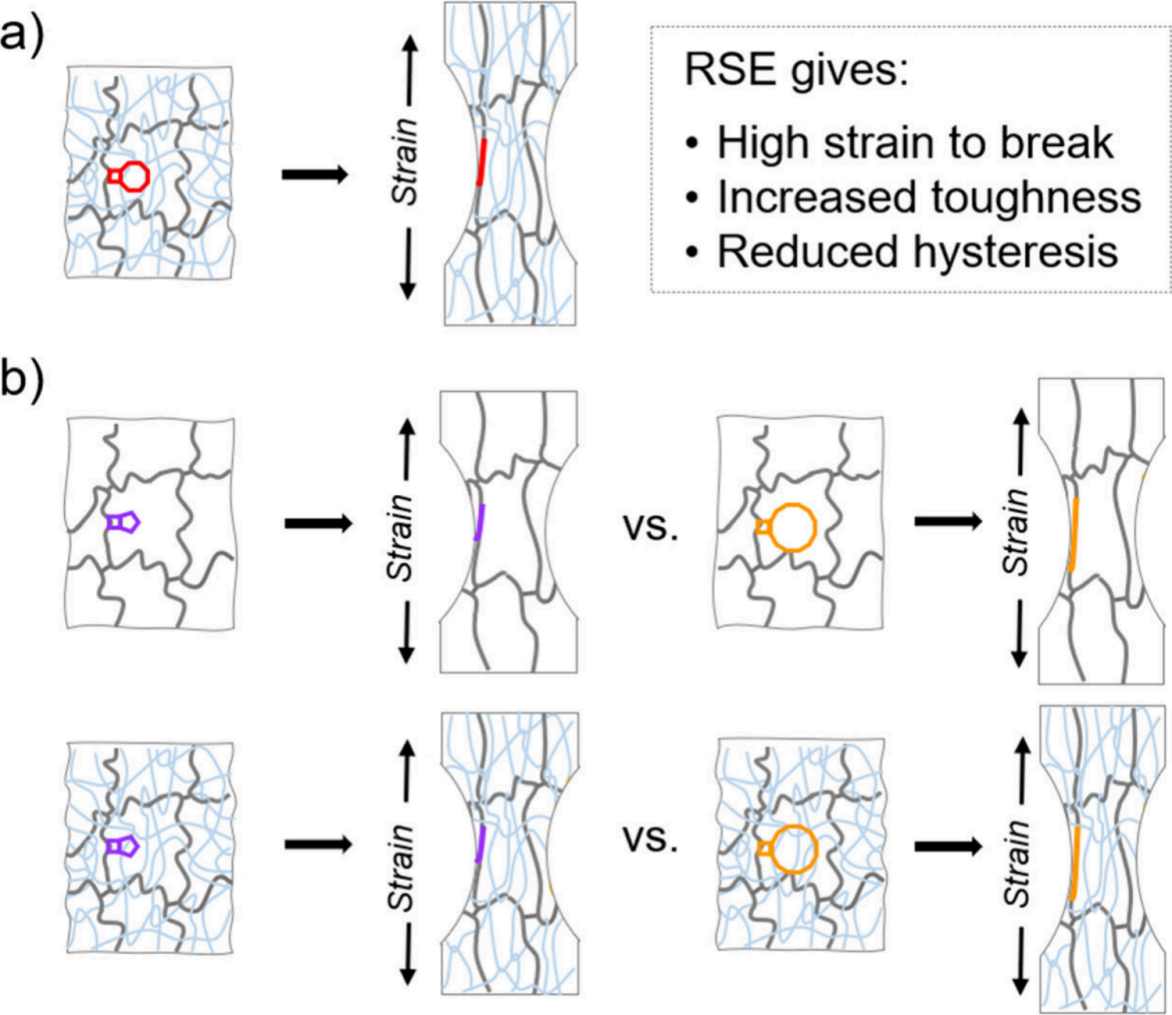
Concept of force-coupled RSE in polymer networks. (a) Schematic of the RSE concept and its impact on mechanical properties relative to programmed chain scission. (Adapted with permission from ref 14. Copyright 2021 AAAS.)^14^ (b) Questions addressed in this work include whether RSE effects extend to single network gels and to what extent the magnitude of RSE impacts properties in both single and double networks.

Here, we address two previously unanswered questions regarding the RSE effect. First, does the RSE effect translate to single networks? Second, to what extent (if at all) is the macroscopic enhancement in strain at break sensitive to the magnitude of RSE at the single strand level (Figure 1b)? We address these questions through a hierarchical experimental design, in which we vary the RSE response in mechanophores and characterize the impact of that response first in individual strands, then in single network gels, and finally in the prestrained first network of double-network hydrogels. The fundamental insights obtained from molecule-to-material correlations inform mechanochemical strategies for changing the high strain behavior and physical limits of polymer networks, independently of their low strain properties.

## Result and Discussion

### Linear Polymers

We began by synthesizing and characterizing the properties of individual strands made with the RSE repeats. The molecular design is shown in Figure 2. Strand extension is provided by the carboxylic acid of bicyclo[3.2.0]­heptane (BCH) and bicyclo[10.2.0]­tetradecane (BCTD) mechanophores,^15^ which react by means of a force-coupled [2 + 2]­cycloelimination to release stored length. Briefly, random copolymers **P**_**5**_ and **P**_**12**_ (numbered to indicate the number of atoms in the fused ring of the repeat unit that provides the extension) were formed through the radical addition copolymerization of the corresponding bicyclic cyclobutene carboxylic acid monomers **m**_**5**_ and **m**_**12**_ in dimethyl sulfoxide (DMSO). Mechanophore reactivity was quantified using single-molecule force spectroscopy (SMFS) implemented through an atomic force microscope (AFM).^16,17^ Random copolymerization of **m**_**5**_ or **m**_**12**_ with 2-acrylamido-2-methylpropanesulfonic acid sodium salt **a** (NaAMPS, comonomer for polymer network) and acetoacetate methacrylate **b** provided linear copolymers **P**_**5**_ or **P**_**12**_, respectively, with **m**:**a**:**b** ≈ 0.27:0.63:0.1 (subscripts *m*, *n*, and *o*, respectively, Figure 2a). The incorporation of **b** is motivated by previous work indicating that it promotes strong attachment of the polymer to the AFM tip.^14^ That attachment point defines the mechanically active region of the polymer in the SMFS experiment, and we account for the variability in its position within the polymer chain by normalizing the force–extension curves, as is standard within the field. Molecular models show that each cycloelimination in **P**_**5**_ extends its contour length by 5.9 Å, whereas the extension per event in **P**_**12**_ is 15.2 Å (Figure 2b). The expected RSE behavior of the polymers was observed in SMFS experiments. Strand lengths from 45 to 175 nm were trapped between a silicon surface and the SMFS probe tip and subsequently stretched at a rate of 300 nm s^–1^. For both **P**_**5**_ and **P**_**12**_, the force–separation curves show a reproducible release of the stored length (up to ∼44% and ∼85%, respectively, of the initial polymer contour lengths) across a force range of 1500–2000 pN. The transition force observed here is similar to a previously reported RSE mechanophore based on a similarly fused cyclobutane,^14,18^ and the shape of the curves are very similar to computational simulations (Figure S9). The observed extensions in the SMFS are close to but less than the maximum theoretical extensions of **P**_**5**_ and **P**_**12**_, which we estimate to be 58% and 150%, respectively (). The theoretical extensions assume quantitative conversion to the more extended *E*-alkene isomers of the product and a statistical incorporation of the RSE monomer between the attachment points; we attribute the discrepancy in theoretical and observed extensions to the fact that neither of these assumptions is likely to be completely correct. Strands that reach the transition force often detach before the plateau is complete, and we were not able to observe full extension in **P**_**12**_. The time required to complete the transition in **P**_**12**_ is longer than that in **P**_**5**_, and the premature detachment/scission of **P**_**12**_ is likely responsible for the failure to observe the full extension.

**Figure 2:**
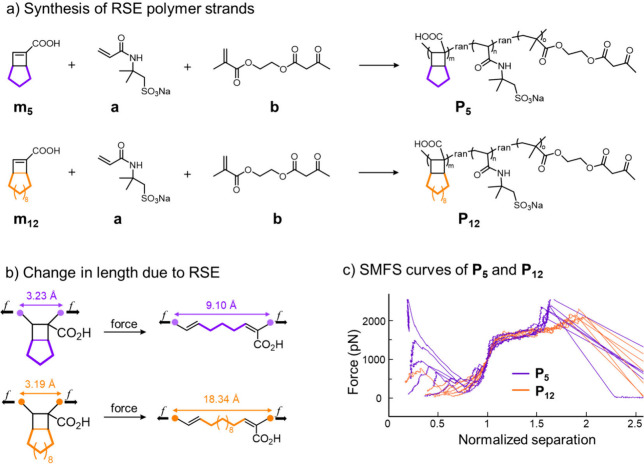
Chemical design and characterization of RSE strands. (a) Synthesis of RSE polymer strands; (b) mechanochemical reaction and associated change in length due to RSE; and (c) representative single-molecule force-spectroscopy (SMFS) curves of linear RSE copolymers **P**_**5**_ and **P**_**12**_ in DMSO.

### Single Network Organogels

The mechanochemically active copolymer strands were subsequently embedded into single network (SN) organogels (Figure 3a). We follow the terminology employed for the linear polymers; i.e., **SN**_**5**_ and **SN**_**12**_ are single networks made using **m**_**5**_ and **m**_**12**_, respectively, as a comonomer with NaAMPS and *N*,*N*′-methylenebis­(acrylamide) (MBAA) as a cross-linker. Details of the network feed compositions are listed in Table 1. SNs are used as synthesized in DMSO. As expected from the similarity in their structures, the ratio of the rate constant for the addition of **m**_**5**_ and **m**_**12**_ at the chain end to the rate constant for the addition of NaAMPS is quite similar (0.41 ± 0.02 and 0.38 ± 0.02, Table S1 and Figure S4).^19^ ^1^H NMR revealed that 98% of **m**_**5**_ and 92% of **m**_**12**_ are ultimately incorporated into linear polymer of **L**_**5**_ and **L**_**12**_ (Figure 3a and Figure S1), respectively, which consist solely of **m**_**5**_ or **m**_**12**_ and NaAMPS in a 3:7 molar ratio. The resulting network structures of **SN**_**5**_ and **SN**_**12**_ are therefore expected to be similar. Consistent with this expectation, we observe that the equilibrium swelling ratios in DMSO (*L*/*L*_0,_ the ratio of longest sample dimension in the fully swollen sample to the as-prepared SNs in DMSO) are 1.92 ± 0.02 and 1.96 ± 0.02, respectively.

**Figure 3:**
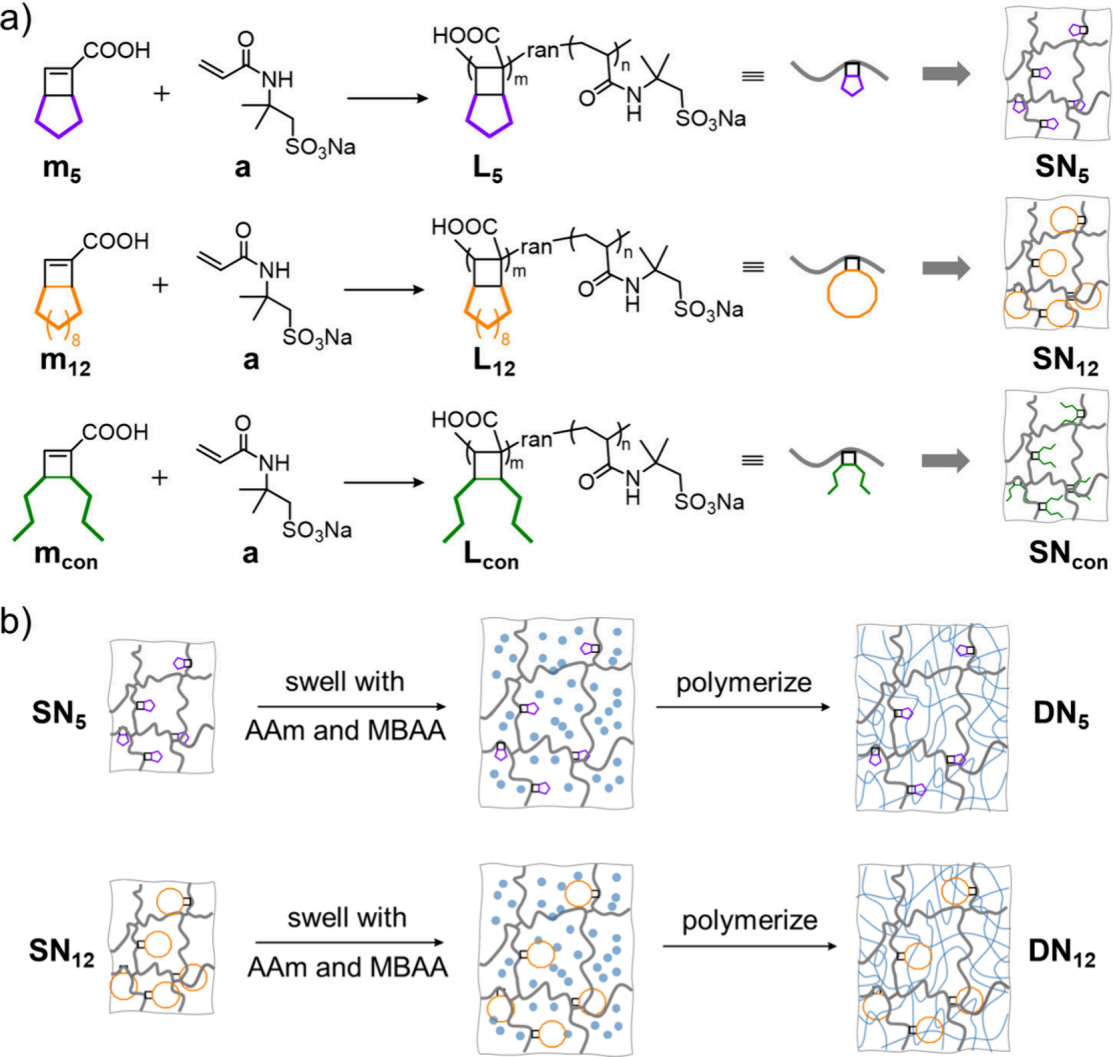
(a) Incorporating RSE into single network organogels. (b) Incorporating RSE into DN hydrogels.

**Table 1:** Feed Concentration of Single Network Organogels and Double Network Hydrogels

	first network						second network	
	**m**_**5**_ (M)	**m**_**12**_ (M)	**m**_**con**_ (M)	AA (M)	NaAMPS (M)	MBAA (M)	AAm (M)	MBAA (M)
**SN**_**5**_	0.6	-	-	-	1.4	0.1	-	--
**SN**_**12**_	-	0.6	-	-	1.4	0.1	-	-
**SN**_**con**_	-	-	0.6	-	1.4	0.1	-	-
**DN**_**5**_	0.6	-	-	-	1.4	0.1	2.5	2.5 × 10^–4^
**DN**_**5‑med**_	0.4	-	-	0.2	1.4	0.1	2.5	2.5 × 10^–4^
**DN**_**5‑low**_	0.2	-	-	0.4	1.4	0.1	2.5	2.5 × 10^–4^
**DN**_**12**_	-	0.6	-	-	1.4	0.1	2.5	2.5 × 10^–4^
**DN**_**12‑med**_	-	0.4	-	0.2	1.4	0.1	2.5	2.5 × 10^–4^
**DN**_**12‑low**_	-	0.2	-	0.4	1.4	0.1	2.5	2.5 × 10^–4^

Single network organogels made from different stored length CB mechanophore monomers, **m**_**5**_ and **m**_**12**_, were used to study the RSE effect. The non-RSE monomer 3,4-dipropylcyclobut-1-ene-1-carboxylic acid (**m**_**con**_) was introduced into the control networks. Wang et al. previously showed that the cyclobutanes along the polymer strand that result from **m**_**con**_ incorporation act as scissile mechanophores,^14^ and so the strands break when stretched to the point that RSE would begin in polymers made from **m**_**5**_ or **m**_**12**_. The SNs containing **m**_**5**_, **m**_**12**_, or **m**_**con**_ were individually fabricated in DMSO due to their low water solubility, and the as-formed DMSO gels were characterized after preparation. Gels of **SN**_**5**_, **SN**_**12**_, and **SN**_**con**_ were cut into 5 mm diameter cylinders with a 1.6 mm thickness. The brittleness of the SN gels made tensile tests impractical (they break frequently when clamping), and so compression tests were conducted to compare the mechanical properties and failure of the networks. During compression, the behavior of the gels was recorded using a custom-built setup. This setup enables observation of the sample behavior through a side-mounted camera and a mirror system (Figure 4c). The onset of cracking was observed visually before the significant change in the stress–strain curve. Therefore, we used the time point corresponding to initial crack formation to assign the failure strain, rather than relying solely on force measurements. This approach was applied consistently across all samples to ensure comparability of strain measurements. Representative compression videos are included in the Supporting Information. We conducted compression tests on *n* = 3 independent replicates for each network formulation (**SN**_**con**_, **SN**_**5**_, and **SN**_**12**_). The corresponding stress–strain curves are shown in Figure 4b, with three individual curves plotted for each sample.

**Figure 4:**
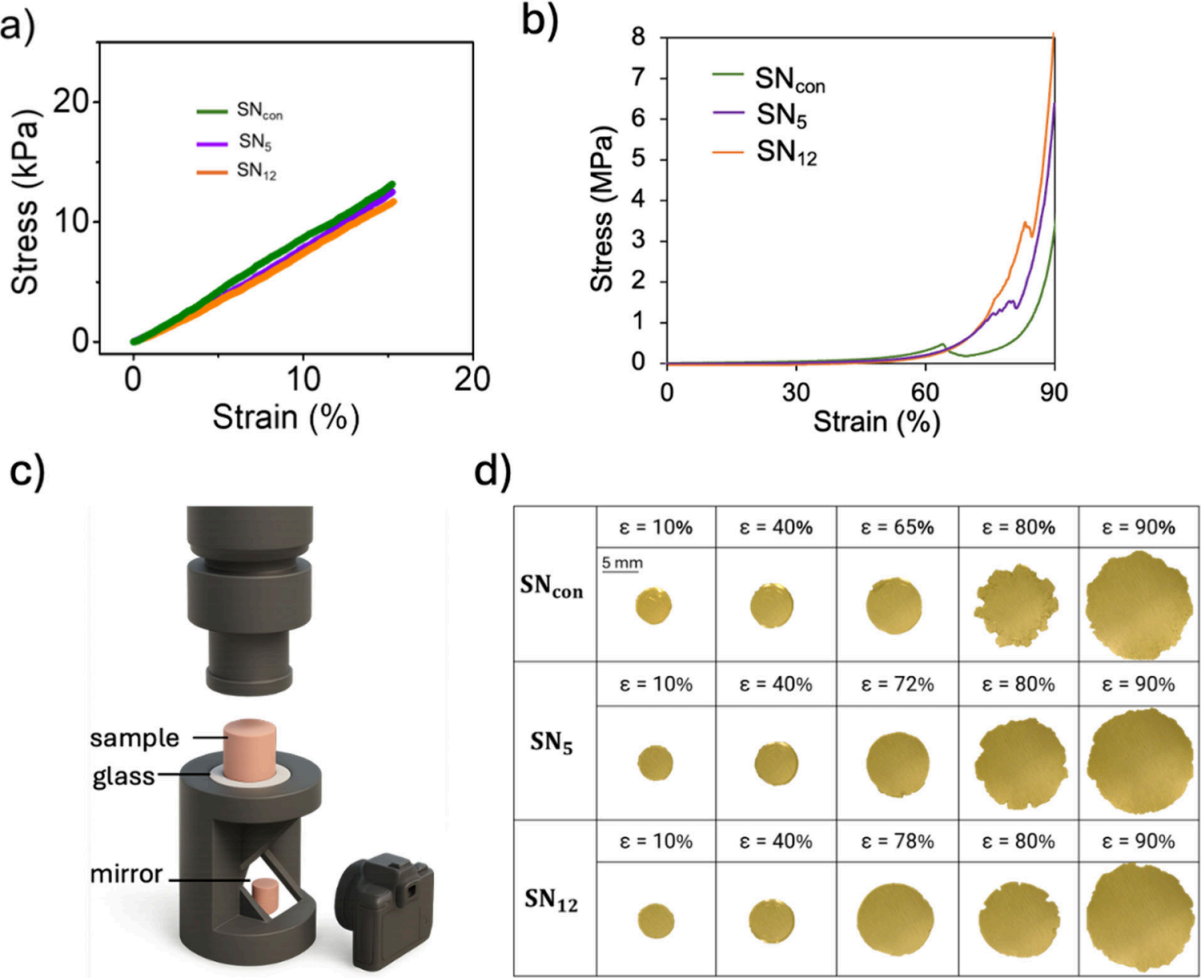
Response of SNs to compression. (a) Strain–stress curves of **SN**_**con**_, **SN**_**5**_, and **SN**_**12**_ in small strain range (*z*-axis compressive strain, 0–15%) and (b) in large strain range (0–90%). Note that the material is not fully expelled from the geometry after rupture, resulting in additional stress recorded after the breaking point. (c) Visual of the compression setup. (d) Photographs of various organogels subjected to different *z*-axis compressive strain, ε_*z*_. The cylindrical SN samples tested had a diameter of 5 mm and a thickness of 1.6 mm.

As shown in Figure 4a and Table 2, the similarity in the initial moduli of these three SNs is consistent with the similarity observed in their swelling behavior: 83 ± 0.5 kPa for **SN**_**5**_, 79 ± 0.4 kPa for **SN**_**12**_, and 85 ± 0.3 kPa for **SN**_**con**_, consistent with the expectation of a comparable network structure prior to deformation. Failure strain was determined as the point at which visible cracking first occurred, identified in real time by using the side-mounted camera and mirror system (Figure 4c). Cracking consistently appeared at a compressive strain of 63 ± 2% in **SN**_**con**_, whereas **SN**_**5**_ and **SN**_**12**_ incorporating RSE mechanophores consistently break at higher strains of approximately 72 ± 2% and 78 ± 2%, respectively. Thus, the trends in strain at break were reproducible across the mechanical tests and the video evidence, revealing delayed crack initiation in **SN**_**12**_ compared to that in **SN**_**5**_. This higher failure strain in **SN**_**12**_ correlates with its increased molecular extensibility, as evidenced by the SMFS measurements.

**Table 2:** Young’s Modulus, Nominal Strain, Nominal Stress, Crack Propagation Strain, and Fracture Energy of SN Organogels and DN Hydrogels Films in a Single-Edge Notched Geometry

	relative swelling ratio	*E* (kPa)^b^	ε_b_ ^b^	σ_b_ (MPa)^b^	ε_p_ ^b^	*G*_F_ (J m^–2^)^b^
**SN**_**5**_	-	83	0.89^c^	1.1	-	-
**SN**_**12**_	-	79	1.13^c^	2.1	-	-
**SN**_**con**_	-	85	0.65^c^	0.26	-	-
**DN**_**5**_	2.21^a^	85.5	6.9	0.93	1.5	1040
**DN**_**12**_	2.27^a^	86.2	18.1	0.92	2.1	2200
**DN**_**5‑low**_	1.97^a^	213.6	4.9	1.0	-	-
**DN**_**12‑low**_	2.06^a^	206.1	9.5	1.0	-	-
**DN**_**5‑med**_	2.10^a^	86.2	13.4	0.65	-	-
**DN**_**12‑med**_	2.20	77.5	19.6	0.71	-	-

a Ratio of one-dimension length of the SNs in their fully swollen state in water prior to polymerization of the second network to the as-prepared SNs in DMSO.

b Determined for as-prepared SN organogels or DN hydrogels.

c Radial strain at break, calculated as ε_r_ = (1/(1 – ε_*z*_))^1/2^ – 1; *E*, Young’s modulus; ε_b_, nominal strain at break; σ_b_, nominal stress at break; ε_p_, crack propagation strain; *T*, tearing energy.

For an incompressible network (i.e., volume is constant under deformation), the radial strain ε_r_ and the axial strain ε_*z*_ are related as (1 – ε_*z*_)­(1 + ε_r_)^2^ = 1,^20^ and so the compressive strain can be converted to equivalent transverse tensile strain using the following relationship: tensile strain ε_r_ = (1/(1 – ε_*z*_))^1/2^ – 1. The corresponding radial strains at fracture (ε_b_) are 69%, 89%, and 113%, respectively. These macroscopic behaviors are qualitatively correlated to the single molecule strand behavior. As the three types of strands are stretched, their responses are expected to be indistinguishable up to the point of mechanophore activation. When the strand tension is high enough to trigger cycloelimination, the responses deviate. In **SN**_**con**_, the polymer strand breaks with no further extension after the cyclobutene cycloelimination, whereas in **SN**_**5**_ and **SN**_**12**_, the strands extend, and as observed in Figure 2b,c the strands in **SN**_**12**_ extend further than in **SN**_**5**_. The extension of a given strand at break can be defined as *e*_b_ = *d*_f_/*d*_i_, where *d*_i_ is the end-to-end distance of the strand (for example, in the unstrained hydrogel) and *d*_f_ is the distance in the fully extended strand at its breaking point. If, as expected, the distribution of monomers between cross-linkers and degree of coiling is similar in **SN**_**5**_ and **SN**_**12**_, then the effective *d*_i_ is also the same. As a result, *e*_b_(**L**_**12**_)/*e*_b_(**L**_**5**_) (relative extension of linear strands made with **m**_**12**_ and **m**_**5**_, respectively) is reasonably characterized by using SMFS measurements on **P**_**12**_ and **P**_**5**_. The ratio of normalized final lengths inferred from the SMFS measurements (Figure 2c): *e*_b_(**L**_**12**_)/*e*_b_(**L**_**5**_) ≈ *e*_b_(**P**_**12**_)/*e*_b_(**P**_**5**_) ≈ 1.3. By comparison, the macroscopic radial tensile strain at break, ε_b_, of **SN**_**12**_ is 1.13 and that of **SN**_**5**_ is 0.89, giving ε_b_(**SN**_**12**_)/ε_b_(**SN**_**5**_) = 1.3 (Table 2). Similar agreement is found relative to the scissile control, by assuming scission in the SMFS curves at ∼1400 pN (just prior to the plateau onset, as reported previously): *e*_b_(**P**_**5**_)/*e*_b_(**P**_**con**_) = 1.5, and ε_b_(**SN**_**5**_)/ε_b_(**SN**_**con**_) = 1.4. The relative ratios of the stretch at break of the networks (1 + ε_b_) are 1.29:1.15:1 for **SN**_**12**_, **SN**_**5**_, and **SN**_**con**_, respectively.

We are duly cautious about reading too much into the comparison of the strand and network extension enabled by RSE, because: (i) as mentioned previously, there is ambiguity in SMFS data regarding the final contour length (especially of **P**_**12**_), and *e*_b_(**P**_**12**_) might be slightly larger than is accounted for in the calculation presented here, leading to a slight underestimate of *e*_b_(**P**_**12**_)/*e*_b_(**P**_**5**_); (ii) **P**_**12**_ and **P**_**5**_ used in the SMFS experiments have a slightly lower content of **m**_**12**_ and **m**_**5**_ than is found in their respective network strands, due to the addition of comonomer **b**, which means that *e*_b_(**P**_**12**_)/*e*_b_(**P**_**5**_) also underestimates *e*_b_(**L**_**12**_)/*e*_b_(**L**_**5**_); (iii) although we anticipate that the average strand comonomer composition between cross-links is similar to the average comonomer composition of the trapped **P**_**12**_ or **P**_**5**_ subchain characterized by SMFS, there is no way to independently verify that this is the case. Nonetheless, these are the first direct observations of covalent RSE behavior in single-network systems, decoupled from the stress-transfer effects typically present in double-network materials, and it is clear that the single-molecule reactivity translates from molecular to macroscopic length to a substantial extent.

The effect of RSE is consistent with expectations based on Lake–Thomas theory, which draws a connection between the toughness of a network and the energy stored in individual network strands that are resisting crack propagating at the point where each strand breaks.^21^ The longer the strand, the more energy is stored. In a typical network, the length of the strands that break is generally taken to be the same as the length of the strands in the as-formed networks. In the networks explored here, however, **SN**_**12**_, **SN**_**5**_, and **SN**_**con**_ begin with effectively identical strand lengths, but the strands that ultimately break are quite different in length due to the mechanochemical extension of the strands under highest tension, and greater RSE leads to more strand length in which to store energy. Only a very small fraction of strands undergo RSE and scission, but these are the strands that resist crack propagation. As a result, the capacity for elastic energy to be stored at the front of the propagating crack increases, leading to higher tearing energies.

### Double Network Hydrogels

We next assessed how the impact of molecular RSE on the extensibility of macroscopic single networks translates to DN hydrogels, where the overall mechanics of network extension are more complicated. A DN gel consists of two interpenetrating networks: a “first network” whose strands are more densely cross-linked and strained by swelling and a “second network” whose strands are less densely cross-linked and in a coiled state. The first network is brittle, while the second is soft and stretchable.^22,23^ When the DN is strained, the prestretched strands of the brittle first network carry the majority of the stress and break independently at increasing strain. The strand scission occurs without catastrophic failure of the entire first network, as the local stress concentration created by the breaking of the first network strands is picked up and redistributed by the interpenetrating second network.^23–27^ We therefore investigated how the differences in RSE between **SN**_**5**_ and **SN**_**12**_ would influence the properties of a DN in which the RSE network acts as the brittle and sacrificial first network. **DN**_**5**_ and **DN**_**12**_ were fabricated by transferring their corresponding SNs to water, swelled to equilibrium, and then polymerizing a second, loosely cross-linked acrylamide (AAm) network within the SN (Figure 3b; feed ratios provided in Table 1). The DN hydrogels were subsequently characterized in their as-prepared state. We also prepared DN hydrogels incorporating the control-fused ring mechanophore **m**_**con**_ as a non-RSE control, but despite our care, we found that solvent exchange from DMSO to water induced numerous microcracks into the surface of the first network gel, which we believe creates uncertainty in the interpretation of any subsequent experiments on the double network that would be formed from it. Therefore, the control DN hydrogel was excluded from further analysis. The increased brittleness of **SN**_**con**_ during solvent exchange is consistent with its earlier failure under mechanical strain described in the previous section.

**DN**_**5**_ and **DN**_**12**_ hydrogels have superior mechanical properties in stretchability and tear resistance than their SN analogues, which allows them to be characterized under uniaxial tension (Figure 5 and Table 2). The initial gap (*L*_i_) between the clamps used to stretch the DN was 0.4 cm, strain (ε) is calculated from the gap between the two clamps (recorded by the DMA instrument) as ε = *L*/*L*_i_ – 1, and the stretch rate was set to (*L*_i_/10) s^–1^, or 10% strain per second based on the initial distance. As seen in the SNs, **DN**_**5**_ and **DN**_**12**_ have effectively identical moduli and low strain extensional behavior (ε < 200%, Figure 5a,b). The ultimate stretch before breaking, however, is significantly greater for **DN**_**12**_ than for **DN**_**5**_ (1809 ± 178% and 690 ± 60%, respectively; see Figure 5a). The dramatic difference in strain at break, ε_b_, is correlated to a change in the mechanism of material failure. High-performance DN gels exhibit yielding accompanied by necking during uniaxial tensile deformation. This yielding behavior, however, is not evident in **DN**_**5**_, which instead shows a continuing increase in stress up to failure, without the distinct plateau stress that is characteristic of necking (Figure 5a). By comparison, **DN**_**12**_ shows clear yielding behavior. These observations suggest the following picture. **DN**_**5**_ and **DN**_**12**_ behave identically until they reach the critical strain where cycloelimination begins. At this point, the first network in both gels softens as the hidden length is released. This initial softening effect is expected to be comparatively weaker in **DN**_**5**_ than in **DN**_**12**_, as a result of the smaller hidden length in the mechanophores of **DN**_**5**_. Consequently, **DN**_**5**_ demonstrates higher stress resistance against further increases in strain compared to **DN**_**12**_ (Figure 5a). This, in turn, leads to more extensive cycloelimination and bond breaking within **DN**_**5**_. This explains the larger hysteresis (Figure 5c) and greater fractional loss of Young’s modulus (Figure 5d) observed for **DN**_**5**_.

**Figure 5:**
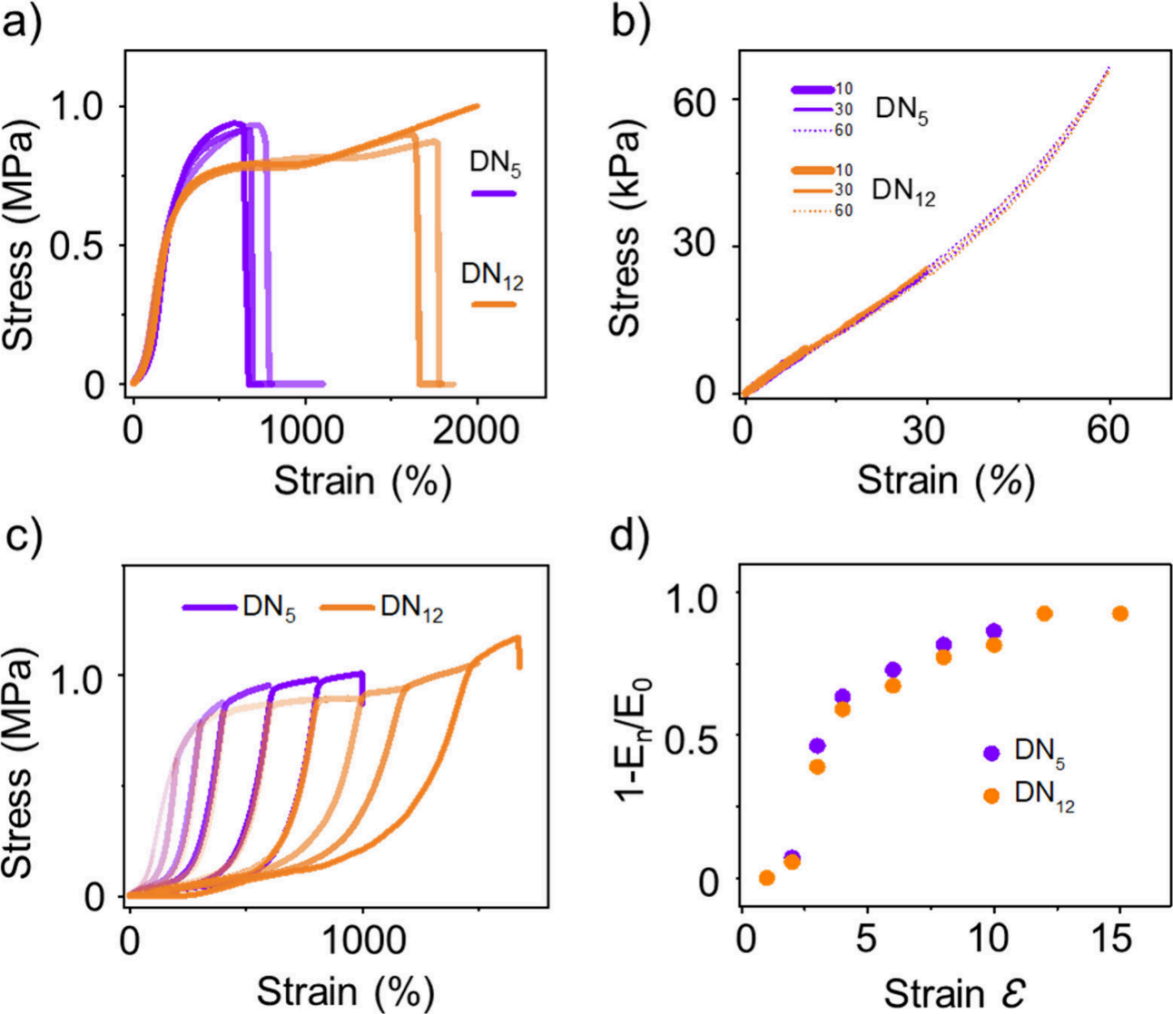
Response of DNs to uniaxial tension. (a) Representative stress–strain curves of unnotched **DN**_**5**_ and **DN**_**12**_; (b) cyclic loading–unloading of **DN**_**5**_ and **DN**_**12**_ in small strain range (0–60%) and (c) in high strain range (0–1670%); and (d) fractional loss of Young’s modulus of **DN**_**5**_ and **DN**_**12**_ as a function of strain, where moduli are calculated from the initial slope of the loading curves. The dimensions of the characterized region of the samples are (4 mm (*h*) × 3 mm (*w*) × 1.6 mm (*t*)).

Furthermore, the softening of the first network—induced by the release of hidden length—effectively enhances the load-bearing capacity of the second network. It appears that the more significant lengthening in **DN**_**12**_ results in a relatively stronger second network, which contributes to the overall DN gel’s greater extensibility.^28^ In contrast, the fracture of the first network **SN**_**12**_ in **DN**_**12**_ initiates at higher strain, at which point a greater fraction of the stress is transferred to the second network, leading to a global discontinuous phase that transfers the load to the second network.^20^ Each strand’s contribution to the elastic modulus is a function of the ratio of its initial (relaxed) end-to-end distance to final contour length.^29^ The introduction of greater RSE preserves the initial end-to-end distance but increases the final contour length, and **DN**_**12**_ therefore reduces a strand’s contribution to the tensile modulus, but that reduction is not as great as in the comparison strand that has shorter RSE, then we observed a smaller decrease in modulus with strain under cyclic loading in **DN**_**12**_ than in **DN**_**5**_.

Hysteresis measurements support the picture of higher levels of strand scission in **DN**_**5**_ compared to **DN**_**12**_. We examined the hysteresis that occurs in sequential loading cycles, where the maximum strain increases with each cycle. Generally, the hysteresis and associated reduction in modulus are attributed to chain scission in the first network. RSE should also reduce the contribution of the extended strand to modulus, but that reduction should be less than that caused by chain scission (a lengthened strand will still contribute to modulus; a broken strand will not). We expect that, at a given strain, more strands break in **DN**_**5**_ compared to **DN**_**12**_, and thus the reduction in modulus at that strain should be greater in **DN**_**5**_ than in **DN**_**12**_. The experimental observations are consistent with this hypothesis, showing that greater RSE helps prevent strand breaking within the first network (Figure 5c,d).^24^ This reduction in strand scission mitigates the fracture event, causing subsequent fractures to occur in different locations and leading to the desired fractal-domain patterns that enhance stretchability and toughness.^30^

The resistance to tearing can be further quantified by the fracture energy (*G*_F_), which represents the energy required per area of crack growth, as determined by Rivlin and Thomas’s method.^31^ The critical stretch for crack propagation in **DN**_**12**_ is 208%, which is 40% greater than the 148% observed in **DN**_**5**_ (Figure 6), and roughly double the *G*_F_ than **DN**_**5**_ of 2200 versus 1040 J/m^2^ (Table 2), respectively. The initial end-to-end distance of strands between cross-linkers is expected to be the same for both DNs, but the final contour length of **DN**_**12**_ should be greater than that of **DN**_**5**_, as evidenced by the results of SMFS (Figure 2c) and calculation (Figure S6). As in the single networks, the extension of highly tensioned polymer strands leads to greater stored energy at the crack front and enhanced macroscopic extension of the double network hydrogels, which is reflected in the increased fracture energy.^32^ In the case of double networks, additional contributions are likely. The release of hidden length in the first network of **DN**_**12**_ results in more load-bearing in the second network than in **DN**_**5**_ when compared to the same strain. This coincides with less breaking of the first network. The smaller hysteresis and decrease of modulus in **DN**_**12**_ (see Figure 5c,d and Figure 6) are consistent with this effect.

**Figure 6:**
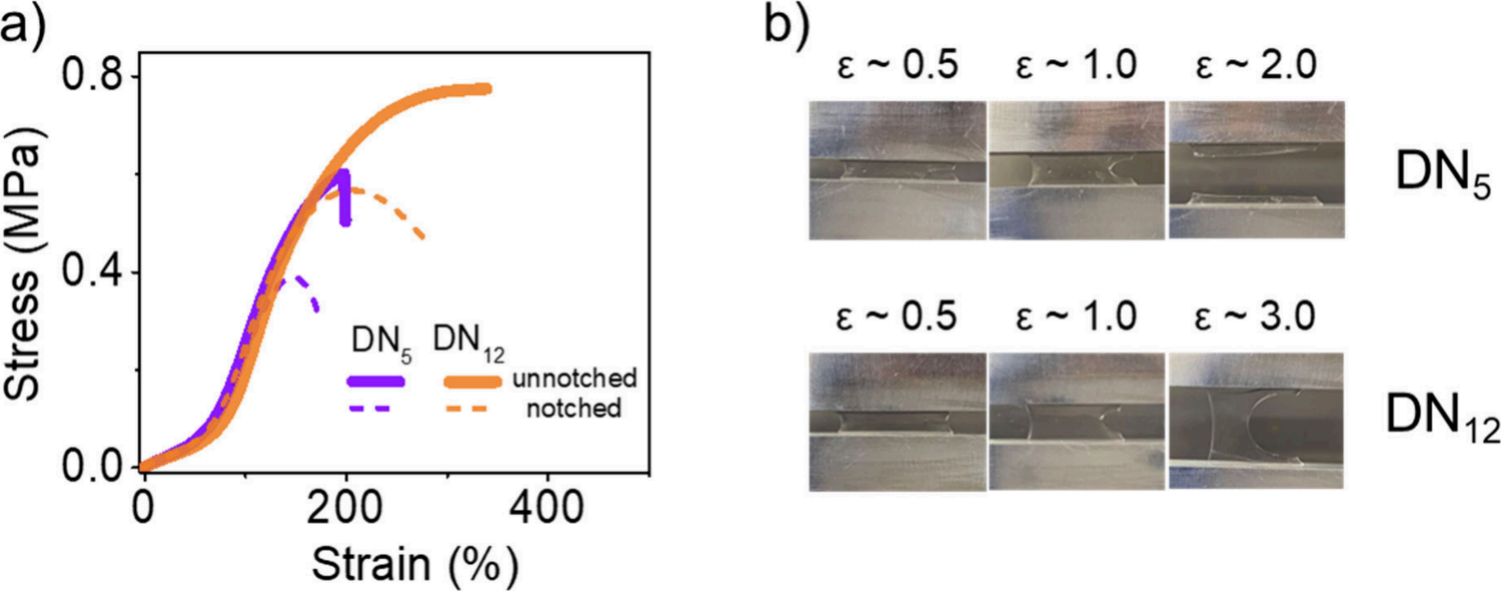
Fracture tests of DNs. (a) The stress–strain curves of unnotched and notched samples of **DN**_**5**_ and **DN**_**12**_. (b) The relative pictures of notched DN samples at different strains were shown. Both stress–strain curves (with and without cracks) before crack propagation are almost identical.

Finally, we tested how the increased RSE translates to other DN compositions. We reduced the overall content of the RSE comonomer by replacing a fraction of the cyclobutene with acrylic acid (AA). Two sets of DNs were made, one in which 1/3 of the mechanophore feed (**m**_**5**_ or **m**_**12**_) was replaced by AA, and one in which 2/3 of the mechanophore was replaced. The resulting DNs are denoted as **DN**_**5‑med**_ or **DN**_**12‑med**_ for the first case, and **DN**_**5‑low**_ or **DN**_**12‑low**_ for the second case, to indicate that they have medium or low mechanophore content, respectively, relative to **DN**_**5**_ and **DN**_**12**_ (Table 2). Our initial hope was that we might be able to compare the effect of change in RSE across, for example, **DN**_**5‑low**_ to **DN**_**5‑med**_ to **DN**_**5**_, but these three gels all have significantly different moduli that we attribute to the fact that we are swapping out a monomer of one reactivity for a monomer of different reactivity. As expected, however, **DN**_**5‑low**_ and **DN**_**12‑low**_ have the same modulus, as do **DN**_**5‑med**_ and **DN**_**12‑med**_ (Figure 7b), enabling us to verify the impact of the RSE ring size within each of the *low* and *med* pairs. As shown in the tensile strain–stress curves of Figure 7a, the ultimate strain for **DN**_**12‑low**_ is greater than **DN**_**5‑low**_ (950% to 490%, see Table 2), and that of **DN**_**12‑med**_ is greater than **DN**_**5‑med**_ (1960% to 1340%). The correlation between mechanophore RSE and network extensibility is consistently observed across all three sets of DN gels originating from the first networks with varying microstructures.

**Figure 7:**
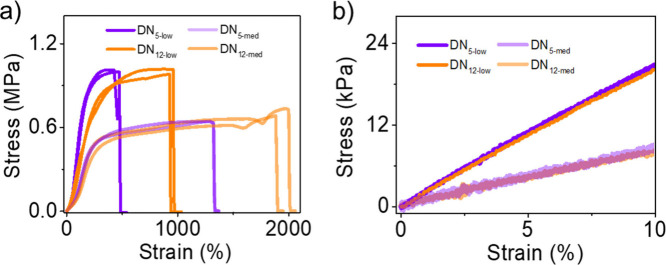
Effect of monomer RSE on the mechanical properties of double networks with different levels of mechanophore content, as determined from tensile tests. (a) Stress–strain curves. (b) Low strain region of panel (a).

## Conclusions

We have observed that changing the capacity for reactive strand extension in single polymer strands delivers the corresponding changes in the extensibility of both single and double network gels that incorporate those strands. That correlation holds consequences for the future development of both sets of networks. First, this work demonstrates that strand extension—previously observed macroscopically only in DN hydrogels—can positively impact the ultimate mechanical properties of much more brittle SNs. Second, the enhancement in macroscopic tensile strain at break is found here to be comparable to the enhancement in molecular strain at break in the individual strands. SNs are far more prevalent materials than DNs, so this work suggests that RSE offers a previously unexploited mechanism for toughening this more common class of materials. Importantly, the enhancement in extensibility and toughness is achieved without changing the modulus of the single network, and RSE therefore offers a molecular mechanism by which modulus and toughness (which are typically anticorrelated) can be tuned independently. On a fundamental level, the combination of tunable RSE and macroscopic mechanical testing offers the potential going forward for a richer set of quantitative molecule-to-material structure–activity relationships to be established. We anticipate that such relationships will prove useful to testing fundamental theories of network fracture mechanisms, and they might also inform methods through which enhanced single network toughness can be systematically and predictively tuned, for example, through the size of the fused ring providing the molecular extension.

A further set of opportunities arises in DNs. As in the SNs, greater RSE in individual strands leads to greater extensibility in the networks, but the relationship is more complex. Perhaps counterintuitively, the addition of a second network to an RSE-containing SN can actually magnify the impact of RSE, because a greater RSE delays fracture in the stress-bearing SN and enables more effective stress redistribution between networks. The changes in stress redistribution mean that the RSE effects can be magnified, as observed here, but we speculate that in other situations, RSE effects might be muted by the second network. For example, DNs in which the ultimate strain is already limited almost entirely by the extensibility of the second network might not see as great an effect from additional RSE in the first network. The ability to systematically tune the behavior of the first network provides an opportunity to probe the mechanisms that govern stress transfer between the two DN components. As an understanding of the structure–activity relationships grows, so too will the ability to dial in changes in ultimate material properties independently of the initial modulus. The behaviors observed here add to a growing menu of molecular, mechanochemical approaches that broaden the combinations of properties available in polymer networks.^32,33^

## Supplementary Material











## Notes

Synthetic procedures for monomers, linear and network polymers; characterization data (^1^H and ^13^C NMR); mechanophore incorporation and reactivity studies; mechanical testing results; CoGEF and molecular dynamics simulation details; BigSMILES strings of linear polymers and single networks (PDF)
Representative compression video of **SN**_**con**_ (MP4)
Representative compression video of **SN**_**5**_ (MP4)
Representative compression video of **SN**_**12**_ (MP4)
Transparent Peer Review report available (PDF)
