# Supplementary material for: Tuning the Ultimate Strain of Single and Double Network Gels Through Reactive Strand Extension
Source: ACS Cent Sci. 2025 Aug 15;11(10):1882–91. doi: 10.1021/acscentsci.5c00932 (PMC12550619; doi:10.1021/acscentsci.5c00932)
Supplement: Supplementary file 1 [file oc5c00932_si_001.pdf]

Supplementary Materials for

Tuning the Ultimate Strain of Single and Double Network Gels Through Reactive Strand Extension

Xujun Zheng[1,2,9], Chun-Yu Chiou[1,2,9], Sunay Dilara Ekim[1,3], Tatiana B. Kouznetsova[1,2], Jafer Vakil[1,2], Yixin Hu[1,2], Liel Sapir[5], Danyang Chen[1,4], Zi Wang[1,2], Michael Rubinstein[1,2,4,6,7], Jian Ping Gong[1,7,8], Nancy R. Sottos*[,1,3], and Stephen L. Craig*[,1,2]

[1]Center for the Chemistry of Molecularly Optimized Networks, Duke University, Durham, NC 27708, USA

[2]Department of Chemistry, Duke University, Durham, North Carolina 27708, USA

[3]Department of Materials Science and Engineering, University of Illinois at Urbana-Champaign, Urbana, Illinois 61801, USA

[4]Department of Mechanical Engineering and Materials Science, Duke University, Durham, North Carolina 27708, USA

[5]Department of Chemistry and the Institute of Nanotechnology and Advanced Materials, Bar-Ilan University, Ramat-Gan, 52900, Israel

[6]Departments of Physics and Biomedical Engineering, Duke University, Durham, North Carolina 27708, USA

[7]Institute for Chemical Reaction Design and Discovery, Hokkaido University, Sapporo 001-0021, Japan

[8]Faculty of Advanced Life Science, Hokkaido University, Sapporo 001-0021, Japan

[9]These authors contributed equally

Email: n-sottos@illinois.edu; stephen.craig@duke.edu

Materials:

All solvents were purchased from VWR or Sigma Aldrich. 2-Acrylamido-2-methyl-1-propanesulfonic acid sodium salt solution (**a**, NaAMPS, 50 wt. % in H_2O), Acrylamide (AAm), acrylic acid (AA), *N*,*N′*-methylenebis(acrylamide) (MBAA), 2-oxoglutaric acid, 2,2-azobis(2-methylpropionitrile) (AIBN), 2-(methacryloyloxy)ethyl acetoacetate (**b**), 2-Hydroxy-4'-(2-hydroxyethoxy)-2-methylpropiophenone (HMP), ethylaluminum dichloride, and methyl propiolate were purchased from Sigma, VWR, or Ambeed and were used as

received unless otherwise noted. AIBN was recrystallized from methanol before use. NaAMPS was precipitated in cold acetone from a 50 wt.% water solution, dried in darkness for use and stored in the freezer.

General methods:

^{1}H and ^{13}C NMR were obtained on the Bruker 500 MHz spectrometer with reference to solvent peak $CDCl_3$ (^{1}H δ = 7.26 and ^{13}C δ = 77.16), DMSO-d_6 (^{1}H δ = 2.55), and D_2O (^{1}H δ = 4.70). All chemical shifts are given in ppm (δ) and coupling constants (J) in Hz as singlet (s), doublet doublet (dd), or multiplet (m). Flash chromatography was conducted on a Teledyne ISCO CombiFlash®200 auto-column system. High-resolution mass spectra were collected on an Agilent LCMS-TOF-DART at Duke University's Mass Spectrometry Facility.

Single-Molecule Force Spectroscopy (SMFS) experiments were conducted in anhydrous DMSO at ambient temperature (~23 °C) in the same manner as described previously using a homemade Atomic Force Microscope (AFM), which comprised of a Digital Instruments scanning head mounted on top of a piezoelectric positioner, similar to the one described in detail previously.[1, 2] Sharp Microlever silicon probes (MSNL), and Silicon Nitride AFM probes (PNP-DB) were purchased from Bruker (Camarillo, CA) and NanoAndMore USA Corp (Watsonville, CA), correspondingly. The spring constants were calibrated for each probe in air, using the MFP-3D system (Asylum Research Group Inc., Santa Barbara, CA), applying the thermal noise method, based on the energy equipartition theorem. Specifically, 20 µL of a 0.1-0.5 mg/mL polymer solution was deposited on a silicon substrate surface and allowed to dry. The silicon substrate was then placed on the piezoelectric stage of the AFM. The sample was then placed on the AFM stage, and the fluid cell was filled with toluene. The system was allowed to equilibrate for approximately 1 hour, or until the cantilever's deflection drift was less than 1 pN/sec, prior to conducting experiments. Force curves were collected in dSPACE (dSPACE Inc. Wixom, MI) and analyzed using Matlab (The MathWorks, Inc., Natick, MA). All data was filtered during acquisition at 500 Hz. After acquisition, the data was calibrated and plotted with software written in Matlab language.

Monomers synthesis:

The synthesis of $\mathbf{m}_{\mathbf{con}}$ was followed by the procedure reported in the literature.[3]

Synthesis of $\mathbf{m}_{\mathbf{5}}$ and $\mathbf{m}_{\mathbf{12}}$:

Compounds **1a**[4] (1.52 g, 10 mmol), or **2a**[4] (2.50 g, 10 mmol), were dissolved in 20 mL MeOH, and the solution was added slowly into the NaOH MeOH solution. The mixture was stirred at room temperature overnight. 40 mL of water was added to the solution, and then MeOH was evaporated from the reaction mixture. The aqueous layer was washed with 3 × 20 mL portions of ethyl acetate, and the aqueous phase was acidified to a pH of approximately 1 - 2 by the addition of 10% aqueous HCl solution. Dilute the mixture with 40 mL of water, and the yellow mixture was extracted with EA. The organic phase was dried over anhydrous $MgSO_4$, filtered, and concentrated. The crude carboxylic acid product was purified by column chromatography (eluent: ethyl acetate/Hexane = 1/9, v/v) to obtain a white powder $\mathbf{m}_{\mathbf{5}}$ (1.2 g, 87.0%), or white powder $\mathbf{m}_{\mathbf{12}}$ (0.86 g, 36.4%).

Characterization data of $\mathbf{m}_{\mathbf{5}}$ and $\mathbf{m}_{\mathbf{12}}$:

$\mathbf{m}_{\mathbf{5}}$: ^{1}H NMR (500 MHz, Chloroform-*d*) δ 11.33 (s, 1H), 6.74 (s, 1H), 3.31 (dd, *J* = 7.2, 3.3 Hz, 1H), 3.05 (dd, *J* = 7.9, 3.3 Hz, 1H), 1.74 (dd, *J* = 13.1, 6.1 Hz, 1H), 1.68 – 1.48 (m, 3H), 1.30 – 1.15 (m, 2H). ^{13}C NMR (126 MHz, Chloroform-*d*) δ 167.25, 150.86, 138.04, 46.63, 44.80, 25.50, 25.32, 22.95. HRMS-ESI (m/z) for $C_8H_{10}O_2$ $(M-H)^-$ Calcd.: 137.0608, Found: 137.0608.

$\mathbf{m}_{\mathbf{12}}$: ^{1}H NMR (500 MHz, Chloroform-*d*) δ 10.55 (s, 1H), 6.79 (d, *J* = 1.1 Hz, 1H), 3.05 – 2.87 (m, 1H), 2.69 (dd, *J* = 10.6, 4.0 Hz, 1H), 1.82 – 1.62 (m, 1H), 1.63 – 1.06 (m, 20H). ^{13}C NMR (126 MHz, Chloroform-*d*) δ 167.65, 152.71, 139.84, 46.16, 45.04, 28.35, 28.30, 27.81, 27.76, 27.35, 27.18, 26.26, 25.74, 23.08, 23.06. HRMS-ESI (m/z) for $C_{15}H_{24}O_2$ $(M-H)^-$ Calcd.: 235.1704, Found: 235.1704.

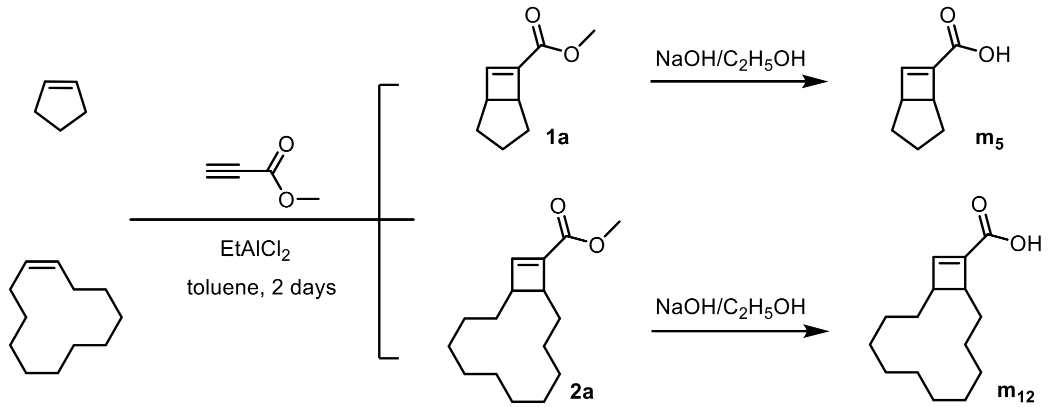

Scheme S1. Synthetic route for monomers $\mathbf{m_5}$ and $\mathbf{m_{12}}$.

Synthesis of linear copolymers P_5 and P_{12} for SMFS:

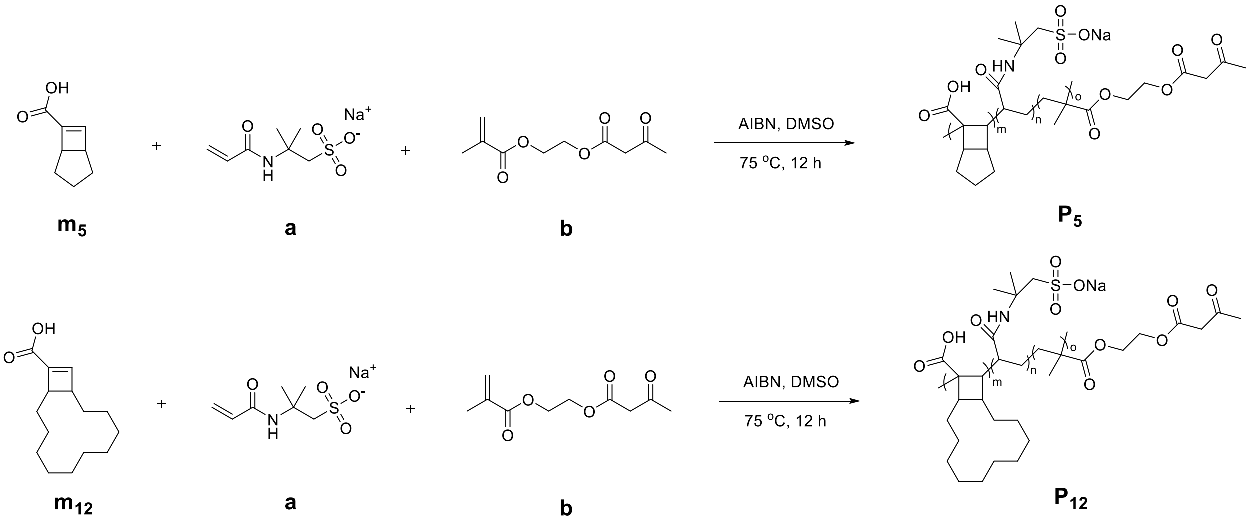

Scheme S2. Synthetic route for polymers $\mathbf{P_5}$ and $\mathbf{P_{12}}$.

2-(methacryloyloxy)ethyl acetoacetate (**b**) was incorporated for strong attachments of the polymer to amine-functionalized AFM tips.[5]

Monomer $\mathbf{m_5}$ (34.5 mg, 0.25 mmol) or $\mathbf{m_{12}}$ (59 mg, 0.25 mmol), co-monomer 2-Acrylamido-2-methyl-1-propanesulfonic acid sodium salt (**a**) (89.2 mg, 0.58 mmol), **b** (19.9 mg, 0.09 mmol) and AIBN (0.3 mg, 1.83 μmol) were dissolved in 0.5 mL DMSO in a 10 mL dried scintillation vial. The mixture was degassed by three cycles of freeze-pump-

thaw. The polymerization was performed at 75 °C overnight, and the resulting viscous solution was slowly bubbled with air for 2 min and then precipitated in acetone. The obtained yellow polymer was then dissolved with 0.5 mL MeOH, and precipitation in acetone was repeated twice more. The incorporation ratio of these two isolated polymers was then calculated to be 0.27:0.63:0.1 for **m**:**a**:**b**, which is ready for SMSF analysis.

Incorporation of $\mathbf{m_5}$ and $\mathbf{m_{12}}$ into $\mathbf{L_5}$ and $\mathbf{L_{12}}$

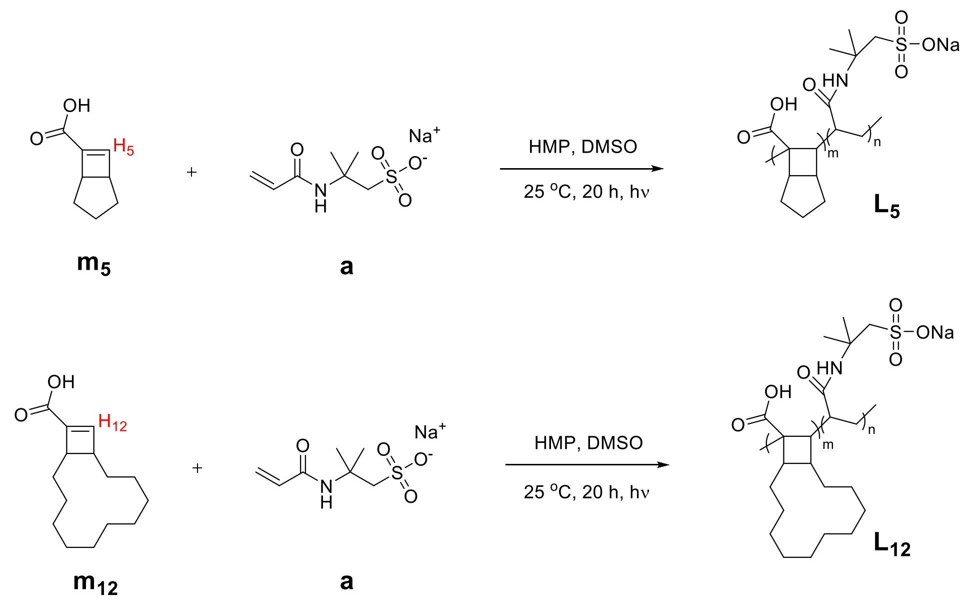

Scheme S3. Synthetic route for polymers $\mathbf{L_5}$ and $\mathbf{L_{12}}$.

Monomer $\mathbf{m_5}$ (41.4 mg, 0.3 mmol) or $\mathbf{m_{12}}$ (70.8 mg, 0.3 mmol), co-monomer 2-Acrylamido-2-methyl-1-propanesulfonic acid sodium salt (**a**) (160.3 mg, 0.7 mmol), internal standard o-dichlorobenzene (10 μL), and photo-initiator HMP (1 mol%, 1.54 mg, 10 μmol) were dissolved in 0.5 mL DMSO-d_6 in quartz NMR tubes. The mixture was degassed by three cycles of freeze-pump-thaw. The polymerization was carried out under 365 nm UV irradiation at 25 °C for various durations (0, 1, 2, 4, 9, 20 h), and the reaction progress was monitored by ^{1}H NMR spectroscopy by integrating the residual monomer peaks relative to an internal standard (Figure S1). The ultimate incorporation of $\mathbf{m_5}$ and $\mathbf{m_{12}}$ into $\mathbf{L_5}$ and $\mathbf{L_{12}}$ is calculated to be 98% and 92%, respectively. Therefore, the resulting network structure of $\mathbf{SN_5}$ and $\mathbf{SN_{12}}$ is therefore expected to be similar.

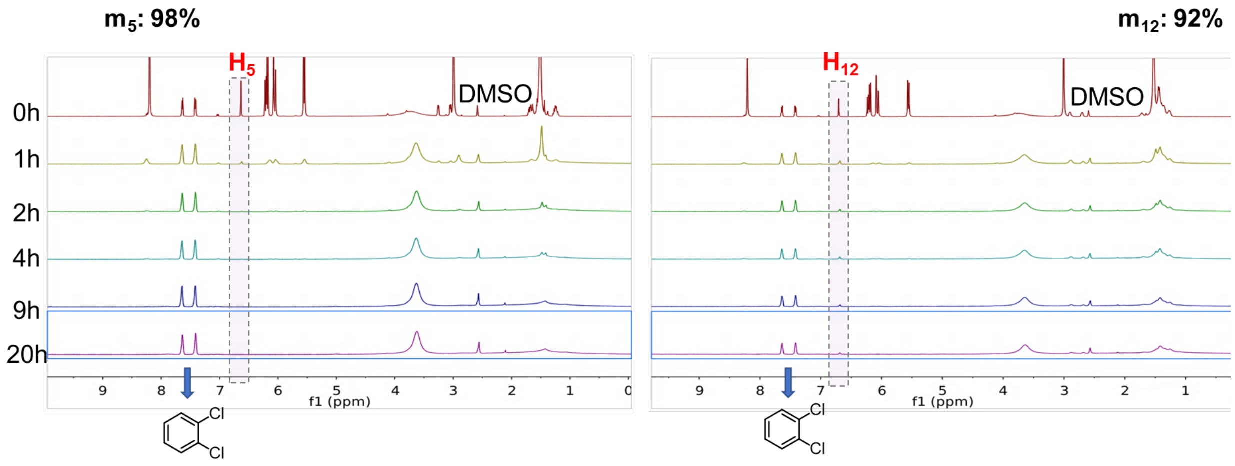

Figure S1. Time-dependent ^{1}H NMR spectra of the polymerization reaction under 365 nm UV irradiation at 25 °C. The reaction progress was monitored by tracking the decrease in the signal intensity of the vinyl proton (peak $\mathbf{H_5}$ and $\mathbf{H_{12}}$, δ ~6.6-6.8 ppm) relative to the internal standard (o-dichlorobenzene, δ ~7.3 ppm, indicated by a blue arrow). The gradual disappearance of peak $\mathbf{H_5}$ and $\mathbf{H_{12}}$ indicates the consumption of $\mathbf{m_5}$ and $\mathbf{m_{12}}$ over time.

Reactivity study of copolymerization $\mathbf{m_5}$ + NaAMPS and $\mathbf{m_{12}}$ + NaAMPS:

The reactivity study is followed by the previous paper.[3, 6]

Four different feed compositions were used: f_2 = 0.08, 0.25, 0.44, and 0.65, with parameter f_1 defined as f_1 = [NaAMPS]/([$\mathbf{m_5}$] + [NaAMPS]) and parameter f_2 defined as f_2 = [$\mathbf{m_5}$]/([$\mathbf{m_5}$] + [NaAMPS]). In each case, we prepared a solution of 0.1 M of total monomer, 0.01 M *o*-dichlorobenzene as the internal standard, and 1 mol% of HMP in 500 µL DMSO-d_6. The well-mixed solution was transferred into an NMR tube and filled with nitrogen. Compositions (chemical shifts at 5.56 corresponding to alkene proton of NaAMPS and 6.65 corresponding to the alkene proton of $\mathbf{m_5}$) were monitored by ^{1}H-NMR spectra at room temperature. Subsequently, the mixture was shone with UV 365 nm in a UV transilluminator for different periods (3 min, 9 min, 15 min, 30 min, 1 h, and 24 h), and ^{1}H NMR spectra were recorded immediately at each time point (representative stacking ^{1}H NMR spectra of f_2 = 0.25 is shown below Figure S2). The conversion of total monomers was calculated with the decreased percentage of the ratio of integration of peaks at chemical shifts of 5.56 and 6.65 relative to the peak integration of the internal standard (δ 7.43). The reactivity ratio r_{NaAMPS} and r_1 for each system were calculated using the

Fineman-Ross (F-R) method (see Figure S4)[6], in which F_1 and F_2 represent the mole fraction of built-in NaAMPS and **m₅** in the copolymer. The 95% confidence limit gives a reasonable estimation of experimental error and the accuracy of the experimental conditions used to generate the composition data. For data analysis, F_1 and F_2 values corresponding with the conversion of total monomers in the range of 5-10% were selected[6], as shown in the Table S1.

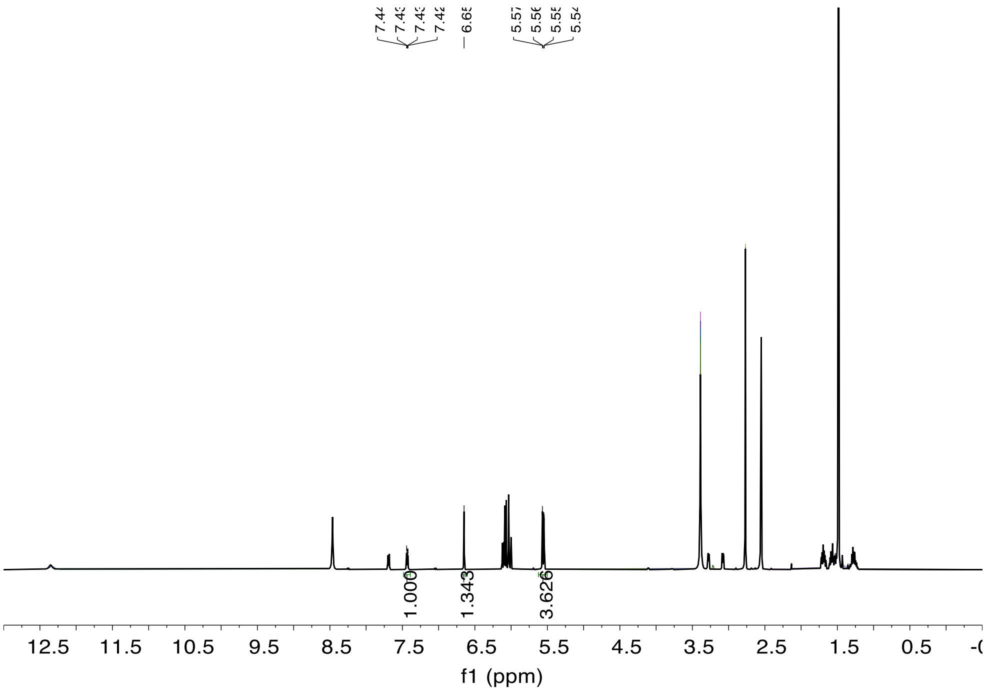

Figure S2. Representative ^{1}H NMR spectra of m_5 + NaAMPS.

For the case of NaAMPS + $\mathbf{m_{12}}$ copolymerization, a similar procedure was conducted with four different feed compositions: f_2 = 0.10, 0.30, 0.50, and 0.70, with parameter f_1 defined as f_1 = [NaAMPS]/([$\mathbf{m_{12}}$] + [NaAMPS]) and parameter f_2 defined as f_2 = [$\mathbf{m_{12}}$]/([$\mathbf{m_{12}}$] + [NaAMPS]). Composition (chemical shifts at 5.56 ppm corresponding to the alkene proton of NaAMPS and 6.72 ppm corresponding to the alkene proton of $\mathbf{m_{12}}$) were checked by ^{1}H NMR spectra at room temperature. The experiment procedure and data analysis are the same as the top paragraph, and the results are shown in Table S1.

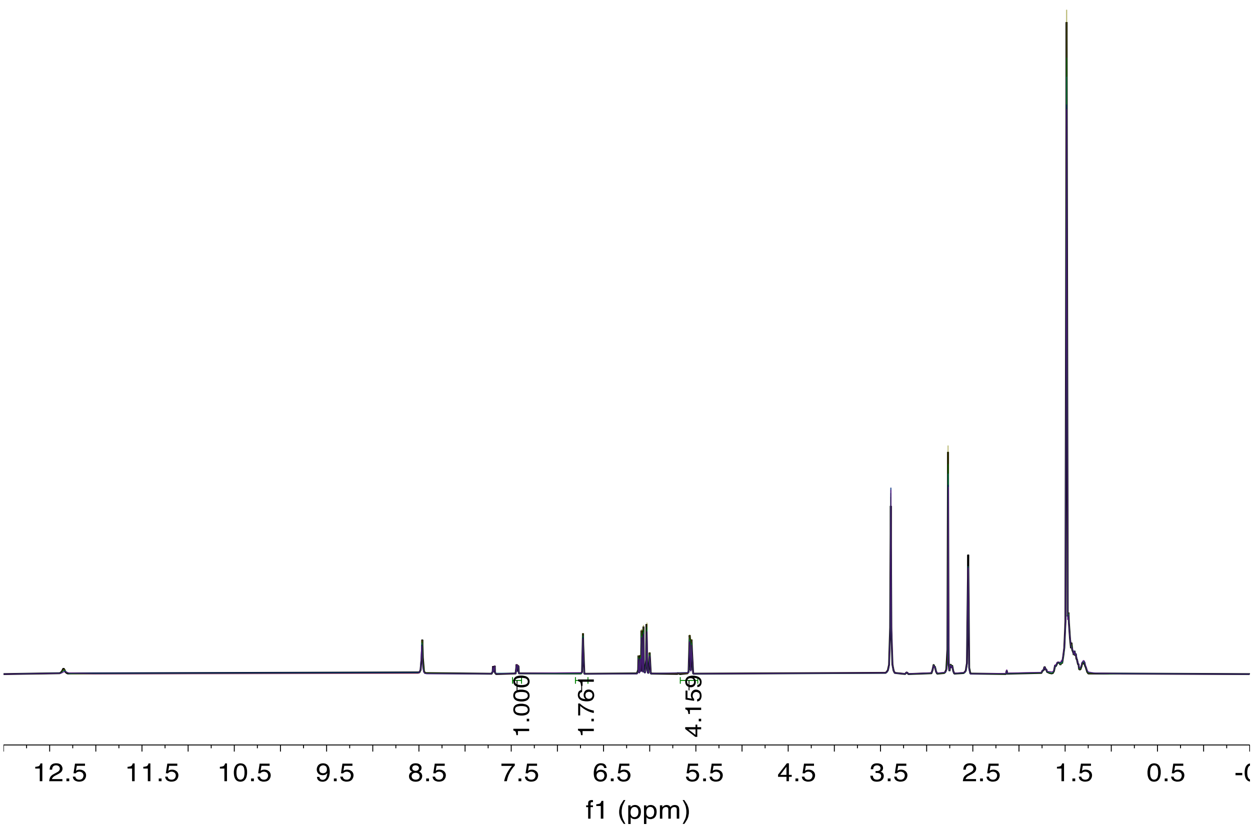

Figure S3. Representative ^{1}H NMR spectra of m_{12} + NaAMPS.

Table S1. Results for copolymerization of NaAMPS + $\mathbf{m_5}$ and NaAMPS + $\mathbf{m_{12}}$: feed ratio f_1 (NaAMPS) and f_2 ($\mathbf{m_5}$ or $\mathbf{m_{12}}$), and built-in ratio F_1 (NaAMPS) and F_2 ($\mathbf{m_5}$ or $\mathbf{m_{12}}$) in the copolymer.

	f_1	f_2	F_1	F_2		f_1	f_2	F_1	F_2
NaAMPS + $\mathbf{m_5}$	0	1	0	1	NaAMPS + $\mathbf{m_{12}}$	0	1	0	1
	0.35	0.65	0.33	0.67		0.3	0.7	0.26	0.74
	0.56	0.44	0.51	0.49		0.5	0.5	0.58	0.42
	0.75	0.25	0.79	0.21		0.7	0.3	0.74	0.26
	0.92	0.08	0.89	0.11		0.9	0.1	0.87	0.13
	1	0	1	0		1	0	1	0

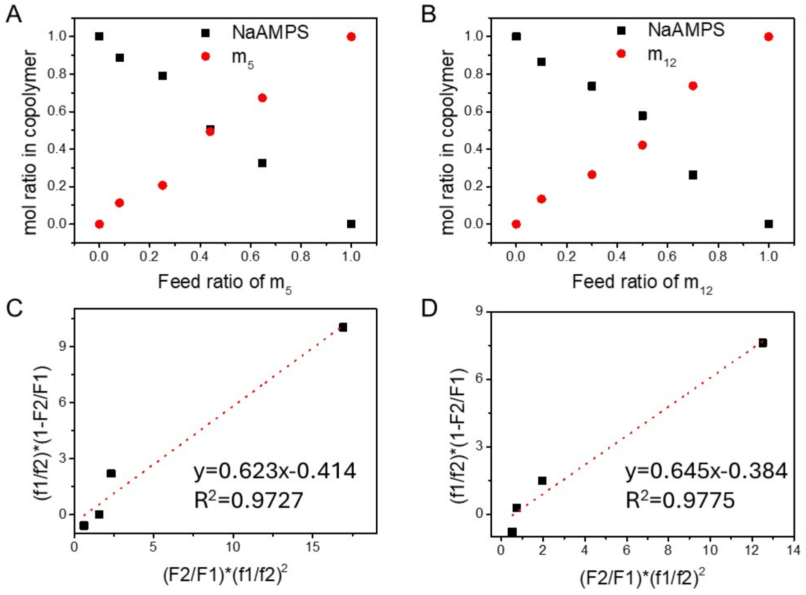

Figure S4. Results of reactivity ratio study of copolymerization of NaAMPS + $\mathbf{m_5}$ and NaAMPS + $\mathbf{m_{12}}$. (A) The built-in ratio of NaAMPS and $\mathbf{m_5}$ in the copolymer as a function of feed ratio of $\mathbf{m_5}$ (conversion of 5%-10% of total monomers was selected). (B) The built ratio of NaAMPS and $\mathbf{m_{12}}$ in the copolymer as a function of the feed ratio of $\mathbf{m_{12}}$ (conversion of 5%-10% of total monomers was selected). (C) The reactivity ratio r_{NaAMPS} (0.62) and r_1 (0.41) calculated using the Fineman-Ross (F-R) method. (D) The reactivity ratio r_{NaAMPS} (0.65) and r_2 (0.38) calculated using the Fineman-Ross method.

Calculation:

COGEF calculations and determination of F_{max} was performed as previously reported via DFT/WB97XD basis set.[7] The ground state geometry was optimized, and its end-to-end distance was constrained and increased with the step increment of 0.1 Å. The maximum force Fmax predicted for each mechanochemical transformation was calculated from the slope between two adjacent points with largest energy difference in the relative energy–relative constrain distance curve. The value of the slope is divided by the Avogadro constant and adjusted to provide force in units of nN (see Figure S5). The similarity in Fmax values for *cis*-$\mathbf{m_5}$ (2.09 nN), *cis*-$\mathbf{m_{12}}$ (2.37 nN) and *trans*-$\mathbf{m_5}$ (2.26 nN), *trans*-$\mathbf{m_5}$ (2.91 nN) indicate similar mechanical reactivity (similar ring opening forces) of BCH and BCTD structures.

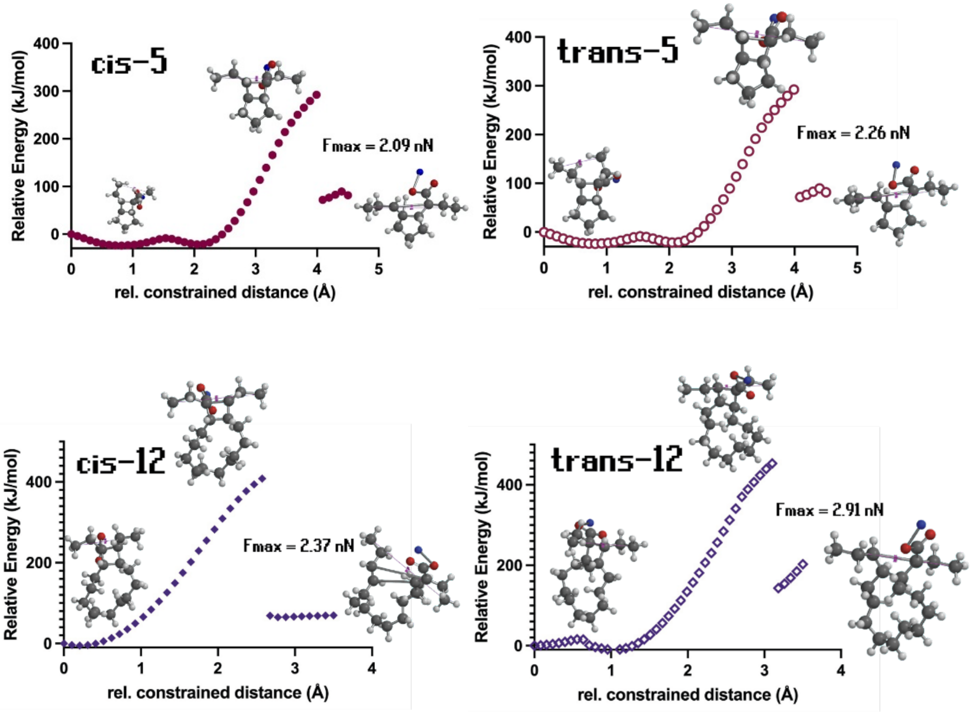

Figure S5. CoGEF modeling for cyclobutane opening. *cis*-$\mathbf{m}_5$, *trans*-$\mathbf{m}_5$, *cis*-$\mathbf{m}_{12}$, and *trans*-$\mathbf{m}_{12}$, respectively, modeled on a polymer backbone. F_{max} was computed by taking the slope between the two points in the energy-displacement curve preceding scission, divided by Avogadro's constant and expressed in nN. The similarity in F_{max} values reflects that the force required for cycloreversion is not significantly dependent on ring size.

CoGEF modeling of polymer contour length increase

The polymer contour length increase study is followed by the previous paper.[3, 6]

The change in polymer contour length before and after transition was modelled by CoGEF using the previous reported method.[2] For the radical copolymerization of BCD and NaAMPS, four isomers of cyclobutane (CB) (see structures *cis*-$\mathbf{m}_5$, *trans*-$\mathbf{m}_5$, *cis*-$\mathbf{m}_{12}$ **and** *tran*-$\mathbf{m}_{12}$ in Figure S6) along the polymer backbone can be formed. For mechanochemical reactions of structures CBs, corresponding alkene products of E and Z isomers are drawn in Figure S6. From ^{1}H NMR, to obtain the exact ratio of *cis*-$\mathbf{m}_5$, *trans*-$\mathbf{m}_5$, *cis*-$\mathbf{m}_{12}$ **and** *tran*-$\mathbf{m}_{12}$ isomers is difficult due to the overlapping and similar chemical shifts of characteristic

proton peaks, here we calculated a range of percent extra extension (EE%) based on the minimum and maximum contour length change. The calculation was performed using molecular mechanics method on the theory level of MMFF (Figure S6).

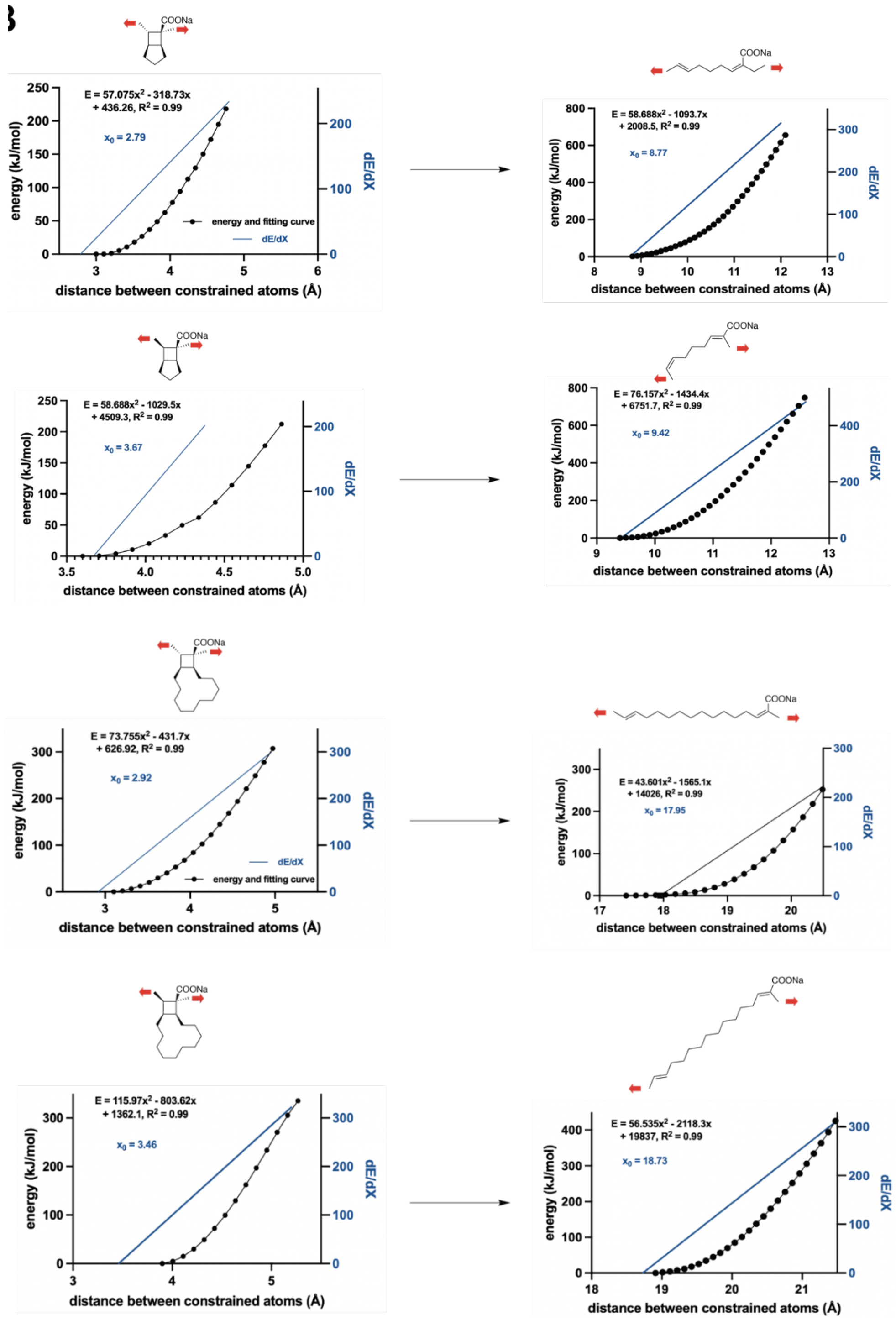

Figure S6. CoGEF modeling for polymer contour length increases. Energy profile of each geometry under applied constrains. First derivative of the quadratic fitting provides a force-distance relation. The equilibrium contour length (x_0) of each structure is calculated by extrapolating force to zero, and x_0 for 12-membered rings is roughly double that x_0 of 5-membered rings. Assuming similar activation values in both **DN₅** and **DN₁₂**, the expected relationship in a polymer network concerning activated strands is **Σ** ($x_{0,\,\mathbf{DN_{12}}}$) **=** 2 **Σ** ($x_{0,\,\mathbf{DN_5}}$), consistent with the observed strain difference between the networks.

$$\frac{L_F}{L_0} = \frac{0.27 \times l_{diene} + 0.63 \times l_{NaAMPS} + 0.1 \times\ l_b}{0.27 \times l_{CB} + 0.63 \times l_{NaAMPS} + 0.1 \times\ l_b}$$

$$EE\% = \left(\frac{L_F}{L_0} - 1\right) \times 100\%$$

L_0 and L_F refer to the initial and final contour lengths of polymer chain; l_{diene} and l_{NaAMPS}, stand for the force-free equilibrium contour length of ring opened CB to diene (for **m_5**, l_{CB} = 3.23 Å, l_{diene} = 9.10 Å; for **m_{12}**, l_{CB} = 3.19 Å, l_{diene} = 18.34 Å) and NaAMPS (l_{NaAMPS} = 2.54 Å; l_b = 2.55 Å).[3,8] The incorporation ratios of **m_5** or **m_{12}**, NaAMPS, and 2-(methacryloyloxy)ethyl acetoacetate (**b**) for **P_5** and **P_{12}** are 27%, 63%, and 10%, respectively, determined from ^{1}H NMR. The range of percentage extra extension (EE%) was calculated to be 58% for **P_5**, and 150% for **P_{12}**, using the above equation.

Synthesis of single networks (SNs)

SN_5, SN_{12}, and SN_{con}: The gels were synthesized by using photoinitiated free-radical polymerization, as previously reported.[3] To synthesize SN gels, **SN_5**, **SN_{12}**, and **SN_{con}** feed concentrations of 0.6 M of **m_5** (41.4 mg, 0.3 mmol), or **m_{12}** (70.8 mg, 0.3 mmol), or **m_{con}** (54.6 mg, 0.3 mmol), 1.4 M of NaAMPS (160.3 mg, 0.7 mmol), 5 mol% (related to total monomers) of cross-linker MBAA (7.7 mg, 0.05 mmol), and 1 mol% (related to total monomers) of HMP (2.24 mg, 0.01 mmol) were dissolved in 0.5 mL HPLC grade DMSO. Each solution was sonicated for 10 mins and then slowly injected into a glass mold consisting of two flat soda-lime glass plates (thickness: 3 mm) separated by silicone rubber (thickness: 0.8 mm) as a spacer. The molds were then transferred into a nitrogen-

filled bag, and each solution was irradiated with a UV transilluminator for 1 h, followed by a UV lamp (365 nm, 4 mW·cm^{-2}) from both sides of the glass mold for 22 h in the glove box. **SN_5**, **SN_{12}**, and **SN_{con}** were characterized as synthesized in DMSO. The codes of gels and reactant concentrations in feed are summarized in Table 1 in the main text.

$SN_{5\text{-low}}$, **$SN_{12\text{-low}}$**, **$SN_{5\text{-med}}$**, **and $SN_{12\text{-med}}$**: The synthesis of **$SN_{5\text{-low}}$**, **$SN_{12\text{-low}}$**, **$SN_{5\text{-med}}$**, and **$SN_{12\text{-med}}$** followed the same procedure described above, with variations in the monomer feed ratios. Specifically, for **$SN_{5\text{-low}}$** and **$SN_{12\text{-low}}$**, 0.2 M of **m_5** (13.8 mg, 0.1 mmol) and 0.4 M of acrylic acid (**AA**, 14.4 mg, 0.2 mmol) were used. For **$SN_{5\text{-med}}$** and **$SN_{12\text{-med}}$**, 0.4 M of **m_5** (27.6 mg, 0.2 mmol) and 0.2 M of acrylic acid (**AA**, 7.2 mg, 0.1 mmol) were used. The codes of gels and reactant concentrations in feed are also summarized in Table 1 in the main text.

Synthesis of double networks (DN_s)

DN_5, **$DN_{5\text{-med}}$**, **$DN_{5\text{-low}}$**, **DN_{12}**, **$DN_{12\text{-med}}$**, and **$DN_{12\text{-low}}$**: The above synthesized SN oganogels **SN_5**, **$SN_{5\text{-med}}$**, **$SN_{5\text{-low}}$**, **SN_{12}**, **$SN_{12\text{-med}}$**, and **$SN_{12\text{-low}}$** were swollen in excessive DMSO and then gradually exchanged with HPLC-grade water (10% increment of water each time). Complete exchange of water typically takes about a week.

To prepare the DN gels, dialyzed SN gels were swollen in precursor aqueous solutions for the stretchable second network containing 2.5 M AAm, 0.01 mol% MBAA, and 0.01 mol% 2-oxoglutaric acid for 2 days (changing the precursor solution every day). Afterward, the swollen gels were sandwiched between two flat soda-lime glass plates and irradiated with a UV lamp overnight in a nitrogen-filled bag. DN gels were characterized as synthesized. The codes of gels and reactant concentrations in feed are summarized in Table 1 in the main text.

Compression tests for SNs

SN_5, SN_{12}, and **SN_{con}** were cut to cylindrical samples with a diameter of 5 mm and a thickness of 1.6 mm. Compression tests of these gels were carried out at the nominal strain rate of 0.5 mm/min in an electromechanical testing frame (Instron) equipped with a 5kN load cell, and the behavior of the gels was recorded using a side-mounted camera in combination with a mirror to capture a top-down view during compression (Figure 4C in the main text). The real-time video was informative, as we noted that the onset of

cracking could be identified visually prior to a deviation in the stress-strain curve. The time point at which visual cracking is observed is denoted as the fracture point and used in subsequent analysis (although the trend using the stress-strain curves is the same). The nominal strain (ε) was measured through the distance between compression grips (recorded by the instrument), and the nominal stress was calculated as the measured load divided by the original cross-sectional area vertical to the load.

Tensile tests and cyclic tests for DNs

Various DN gels $\mathbf{DN_5}$, $\mathbf{DN_{5\text{-}med}}$, $\mathbf{DN_{5\text{-}low}}$, $\mathbf{DN_{12}}$, $\mathbf{DN_{12\text{-}med}}$, and $\mathbf{DN_{12\text{-}low}}$, the dimensions of the characterized region of the samples are (4 mm (h) x 3 mm (w) x 1.6 mm (t)). Uniaxial tensile tests of these gels were carried out at the nominal strain rate of 10%/s. The nominal strain (ε) was measured through the distance between clamps (recorded by the DMA instrument), and the nominal stress was calculated as the measured load divided by the original cross-sectional area vertical to the load.

Cyclic tensile tests of $\mathbf{DN_5}$ and $\mathbf{DN_{12}}$ were performed using the same experimental setup. The samples were first stretched to a certain strain (strain rate: 10%/s) and then immediately unloaded (strain rate: -10%/s). Then, they were stretched to an increased maximum strain and unloaded again. The fraction of modulus loss of $1-E_n/E_0$, in which E_n is the modulus determined from $(n+1)^{th}$ loading curve within a small strain of $\varepsilon < 0.1$, and E_0 is the modulus of 1st loading curve was then calculated (see Figure 5d in the main text).

Tearing test for $\mathbf{DN_5}$ and $\mathbf{DN_{12}}$

Tearing tests were performed on the same instrument with wider clamps.[3] Test pieces were cut to be 20 x 20 mm using a razor blade; the final geometry of the samples loaded was 20 x 4 x 0.8 mm. For notched samples, a 5 mm cut was made with a razor blade perpendicular to the edge of the square. The exact thickness and width of each sample were measured with a caliper before testing. Samples were mounted onto clamps at a gauge length of ~1.5 mm, and then stretched to a force of 0.1 N, which resulted in an initial gauge length of h_0 4 ± 0.5 mm; Tests for both unnotched and notched samples were run at a strain rate of 0.4%/s. The nominal stress of unnotched samples was calculated

as the measured load divided by the original cross-sectional area vertical to the load, while for notched samples, the remaining cross-sectional area: thickness*(original width –5 mm cut length) was applied to calculate the nominal stress. No slippage was detected for the sample data included. Fracture energy, G_F, was calculated using the Thomas-Rivlin method[9] with the equation below:

$$G_F = W(\varepsilon_p)h_0,$$

where $W(\varepsilon_p)$ is the strain energy per unit volume in the region of the test piece which is in a state of pure shear at a given strain. The initial height between clamps, h_0, was recorded as the height when the sample was under 0.1 N force at the start of the test. The strain energy density was obtained by integrating the area beneath the stress strain curve of an un-notched sample up to the critical strain ε_p, at which the notched sample began to propagate (see Figure 6 in the main text).

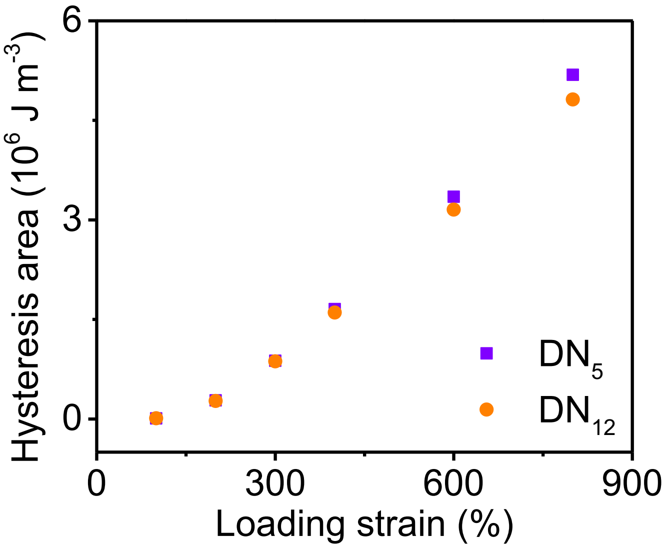

Figure S7. Cumulative hysteresis as a function of loading strain for DN_5 and DN_{12}. The hysteresis is calculated by numerically integrating the stress-strain curves shown in Figure 5c and taking the difference in the loading and unloading curves: $\int_0^{\varepsilon_{app}}(\sigma_1 - \sigma_2)d\varepsilon$, where σ_1 and σ_2 are the nominal stresses during the loading and unloading cycles,

respectively. The hysteresis at a given strain includes hysteresis from all preceding stress-strain curves, and so is the true hysteresis associated with loading/unloading to that strain from the as-formed DN.

Summary of Molecular Dynamics Simulations

Methods

Polymers were simulated by Molecular Dynamics (MD) in an implicit θ-solvent using the Kremer-Grest beads-spring model.[10,11] Non-bonded monomers were interacting via the Lennard-Jones (LJ) potential[12,13]

$$U_{\mathrm{LJ}}(r;r_c) = \begin{cases} 4\varepsilon\left[\left(\frac{\sigma}{r}\right)^{12} - \left(\frac{\sigma}{r}\right)^{6} - \left(\frac{\sigma}{r_c}\right)^{12} + \left(\frac{\sigma}{r_c}\right)^{6}\right] & \square \quad r \le r_c \\ 0 & \square \quad r > r_c \end{cases} \qquad \text{(eq. S1)}$$

where $r_{c,\mathrm{LJ}} = 2.5\sigma$ is the length scale where the LJ interactions are truncated. To simulate an implicit θ-condition we set $\varepsilon = 0.298$.Click or tap here to enter text. Either of two interaction potentials is used to model bonded interactions. *Unbreakable bonds* are modeled by the Finitely Extensible Nonlinear Elastic (FENE) model,[11]

$$U_{\mathrm{FENE}}(r) = \begin{cases} -\frac{1}{2}KR_0^2\, ln\left[1 - \left(\frac{r}{R_0}\right)^2\right] & \square \quad r \le R_0 \\ \infty & \square \quad r > R_0 \end{cases} \qquad \text{(eq. S2)}$$

where the spring constant K is set to $30\ k_{\mathrm{B}}T\sigma^{-2}$, and the cutoff distance R_0 is set to 1.5σ. The total bond interaction for the unbreakable bonds is then set as $U_b(r) = U_{\mathrm{FENE}}(r) + U_{\mathrm{LJ}}\left(r; r_{c,FENE}\right)$, where in the second term $r_{c,\mathrm{FENE}} = 2^{1/6}\sigma$ and $\varepsilon_{\mathrm{FENE}} = 1\ k_{\mathrm{B}}T$.

Breakable bonds are modeled by the Morse potential,[14]

$$U_{\mathrm{Morse}}(r) = D_e\left[1 - e^{-a(r-r_0)}\right]^2 \qquad \text{(eq. S3)}$$

where the dissociation energy is set to $D_e = 90\ k_BT$, the equilibrium length to $r_0 = 0.96$, and $a = 2.35$. The latter two parameters were chosen to keep the equilibrium properties of the breakable and unbreakable bonds as similar as possible, specifically the location

of the potential minimum and the bond stiffness (second derivative the the minimum).

We simulated chains that include very short loops, as illustrated in Fig. S8, where rupture of all breakable bonds results in length doubling of the entire chain. The number of Morse bonds is set to $N = 45,59,74,87,101,112,127,141,154,$ and 175, with $2N + 2$ FENE bonds, $2N + 3$ monomers, and a contour length (before bond scission) of $L = l(N + 2)$, where $l \approx \sigma$ is the average bond length. Assuming a mapping of $\sigma = 1\,nm$, this contour length corresponds to the length range $45 - 175\,nm$ of strands measured by AFM.

All chains were initially equilibrated for $2 \cdot 10^6\,\tau_{LJ}$ in the unperturbed state, where all bonds were parametrized as FENE bonds. Then, the Morse bonds were switched on, and the chains were subjected to extension loading at several stretching velocities, $v = 5.8 \cdot 10^{-6}$, $5.8 \cdot 10^{-5}$, $5.8 \cdot 10^{-4}$, and $5.8 \cdot 10^{-3}\,\sigma/\tau_{LJ}$, by performing small chain extension steps of $5.8 \cdot 10^{-2}\,\sigma$. The average force, f, operating on the end beads was recorded as a function of chain elongation, R.

All molecular dynamics simulations were performed with an integration time-step of $0.01\,\tau_{\mathrm{LJ}}$ and using the Large-scale Atomic/Molecular Massively Parallel Simulator (LAMMPS) package.[15]

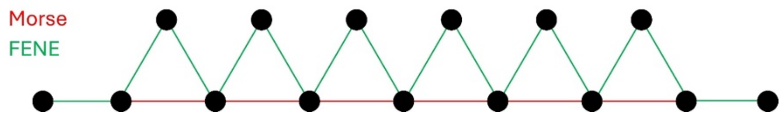

Fig. S8. Scheme of the chain architecture used in the MD simulations. Each chain contains N Morse bonds (red), $2N + 2$ FENE bonds (green), and $2N + 3$ monomers (black circles).

Results

The simulated force-extension curves of the polymer chains are shown in Fig. S9. When stretched to some critical force, f_c, at $R/L \approx 1$, bond rupture begins along with the

ensuing RSE. Bond rupture events show a characteristic "saw-tooth" pattern, where after each scission the tension drops. Decreasing the stretching velocity allows more time for bond scission, which thus occurs at logarithmically lower critical forces (Fig. S9a). At a constant stretching velocity *v*, longer chain length *L* shows very robust force-extension curves, with slightly smaller critical forces, f_c, and smaller tension drops upon scission (Fig. S9b) because longer chains result is lower extension rate *v/L* at the same stretching velocity.

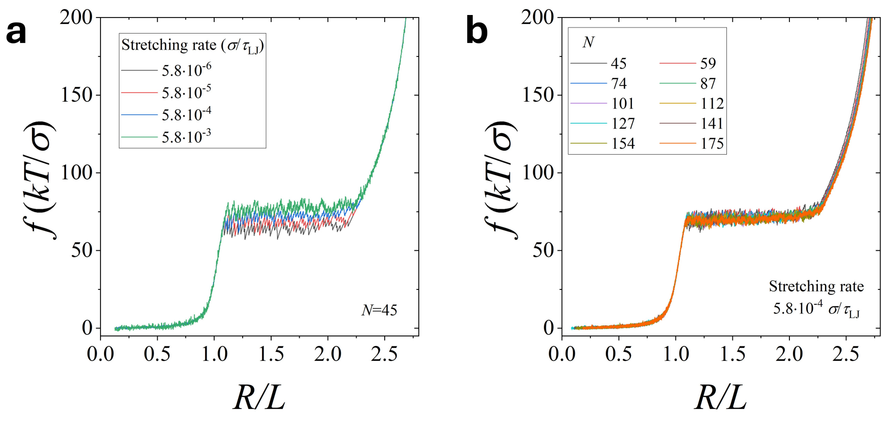

Fig. S9. Force-extension curves from MD simulations. The chain extension is normalized to the contour length of the chain before any bonds were ruptured. **(a)** The force-extension curves of a chain with $N = 45$ at several stretching rates. **(b)** The force-extension curves of several chain lengths at a constant stretching velocity of $5.8 \cdot 10^{-4}\ \sigma/\tau_{LJ}$.

We can map the simulation setup to the experiments shown in the main text, by assuming the bond length $\sigma = 0.15\ nm$ of the Morse potential (corresponding to a C-C bond) and $\tau_{\mathrm{LJ}} = 1\ ps$ (corresponding to the attempt frequency to break the bond). In this case, the extension velocity, v, in simulation that is equivalent to the experimental velocity 300 nm/s would be $\sim 3 \cdot 10^{-10} \sigma/\tau_{LJ}$. The variation of the critical force, f_c, with stretching velocity is shown in Fig. S10. This critical force was estimated by averaging over all peaks in the saw-tooth pattern for each simulated condition. As predicted, this critical force varies linearly with the logarithm of the stretching rate, Fig. S10a. All data collapses onto

a single curve by plotting f_c as a function of the the extension rate, v/L, Fig. S10b. We can approximate $f_c = c \cdot \log[(v/L)/\omega]$, where c and ω are numerical coefficients with unit of force and extension rate, respectively. Through the best fit of the data in Fig. S10b for each N we can extrapolate the critical force to the equivalent experimental rate. For example, for $N = 45$, we find $c = 4.0 \pm 0.2\ k_B T/\sigma$ and $\omega = 2.1 \pm 0.1\ \cdot 10^{-24}\ \tau_{LJ}^{-1}$, and so predict $f_c = 50 \pm 5\ k_B T/\sigma$ at the experimental rate, which corresponds to $1.4 \pm 0.1\ nN$.

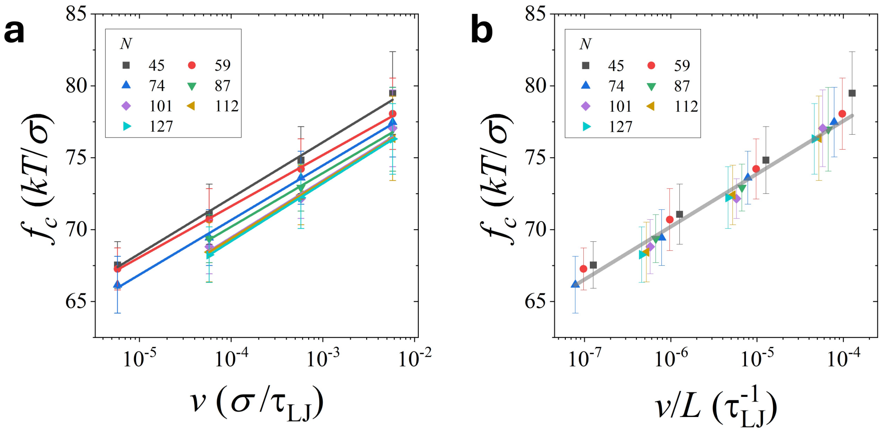

Fig. S10. Dependence of the mechanophore activation tension f_c on extension velocity v for different chain lengths L. **(a)** Longer chains correspond to lower extension rate v/L and therefore lower activation tension. **(b)** Normalizing the x-axis by chain length collapses all the data on a single curve. The gray line is a best linear fit through all the data points.

NMR Spectra

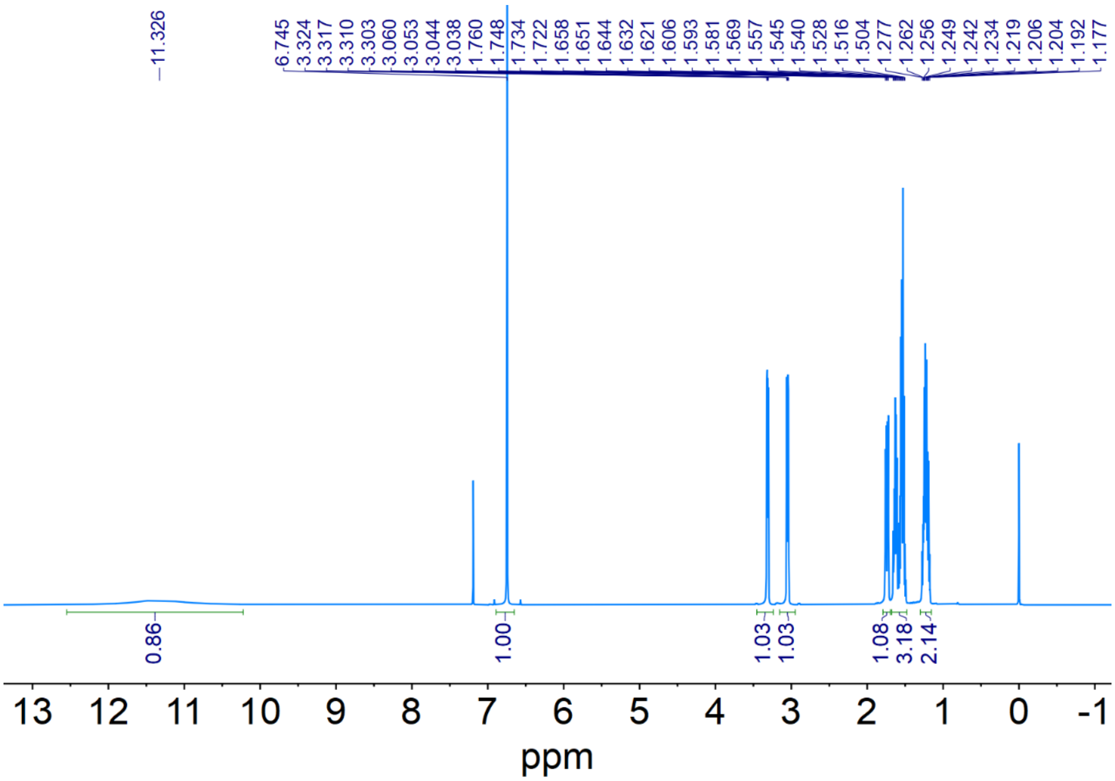

Figure S11. ^{1}H NMR spectrum of **m**$_5$ (500 MHz, 298 K, $CDCl_3$).

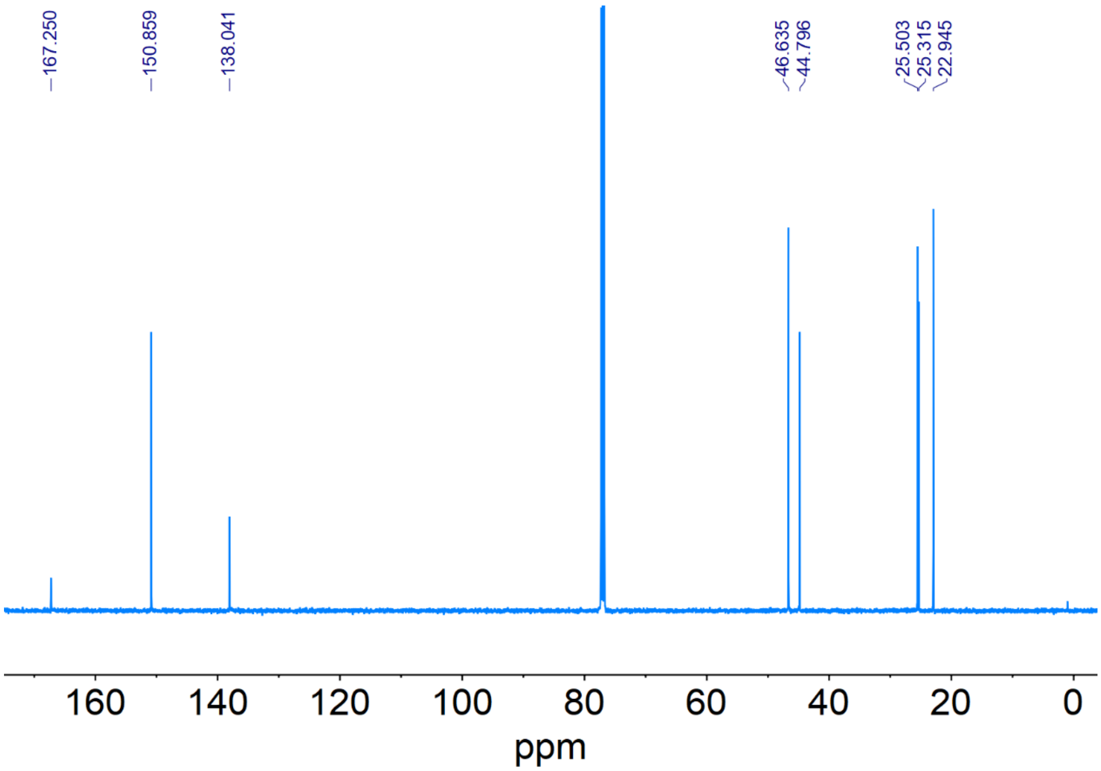

Figure S12. ^{13}C NMR spectrum of **m_5** (500 MHz, 298 K, $CDCl_3$).

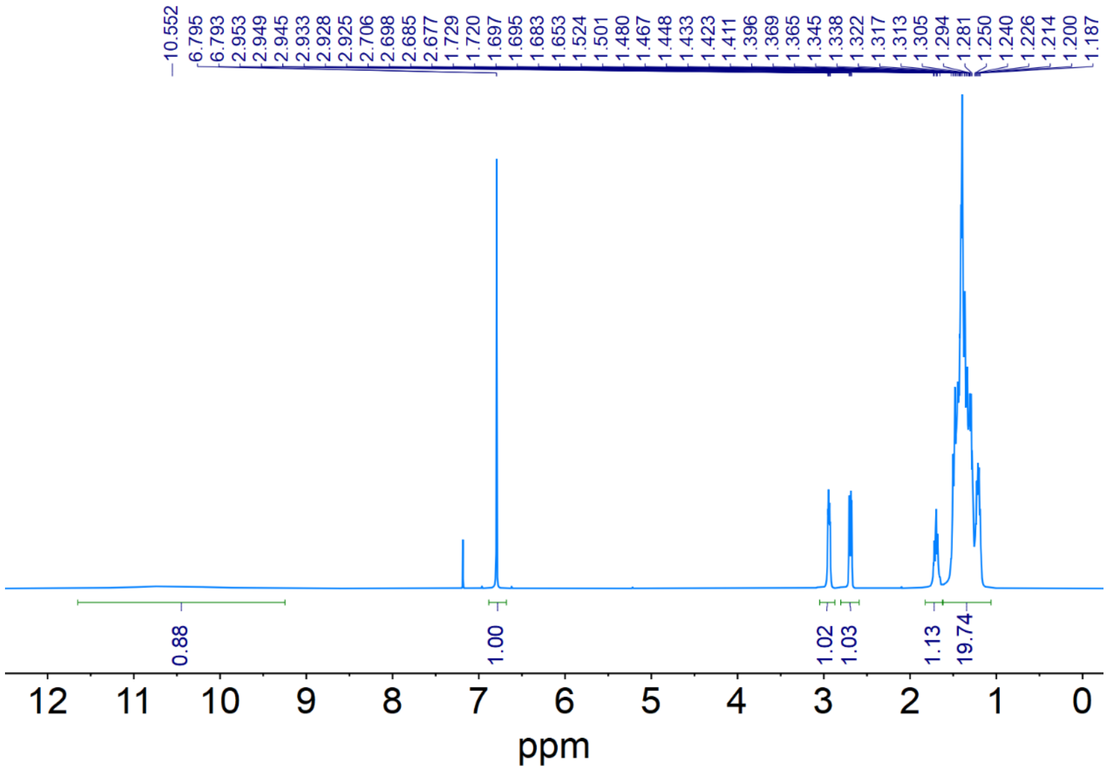

Figure S13. ^{1}H NMR spectrum of **m_{12}** (500 MHz, 298 K, $CDCl_3$).

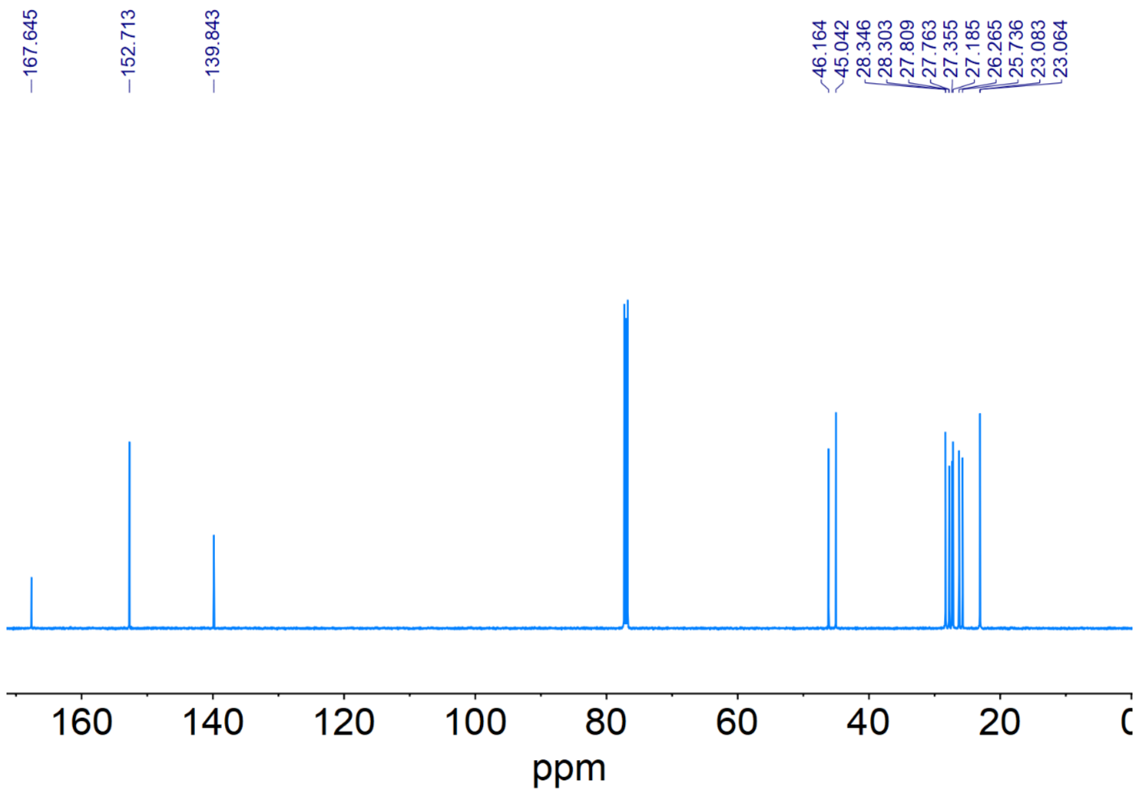

Figure S14. ^{13}C NMR spectrum of $\mathbf{m_{12}}$ (500 MHz, 298 K, $CDCl_3$).

Polymer/ Network	Structure	BigSMILES Representation
**L5**	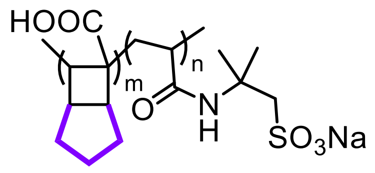	{[][$]C1C(C2C1(CCC2))(C(=O)O)[$],[$]CC(C(=O)NC(C)(C)CS(=O)(=O)[O-])[$].[Na+][]}
**L12**	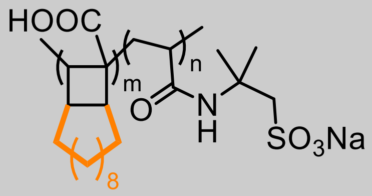	{[][$]C1C(C2C1(CCCCCCCCCC2))(C(=O)O)[$],[$]CC(C(=O)NC(C)(C)CS(=O)(=O)[O-])[$].[Na+][]}
**L8**	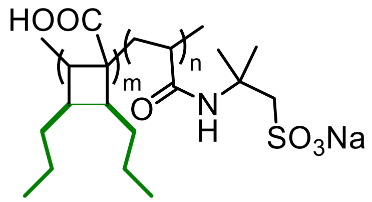	{[][$]C1C(C(CCC)C1(CCC))(C(=O)O)[$],[$]CC(C(=O)NC(C)(C)CS(=O)(=O)[O-])[$].[Na+][]}
**SN5**	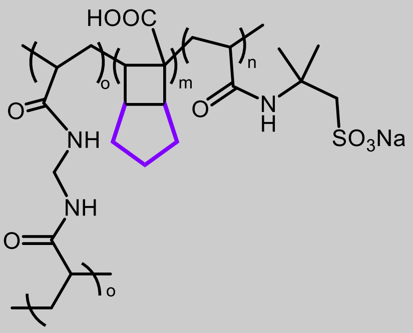	{[][$]C1C(C(=O)NCNC(=O)C=C)[$],[$]C1C(C(=O)NCNC(=O)[$]CC[$])[$],[$]C1C(C2C1(CCC2))(C(=O)O)[$],[$]CC(C(=O)NC(C)(C)CS(=O)(=O)[O-])[$].[Na+][]}
**SN12**	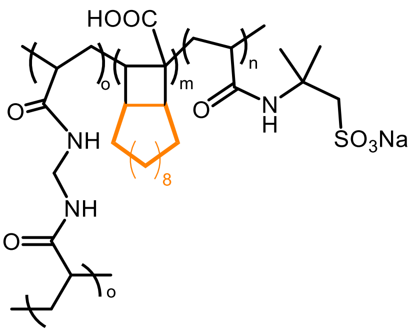	{[][$]C1C(C(=O)NCNC(=O)C=C)[$],[$]C1C(C(=O)NCNC(=O)[$]CC[$])[$],[$]C1C(C2C1(CCCCCCCCCC2))(C(=O)O)[$],[$]CC(C(=O)NC(C)(C)CS(=O)(=O)[O-])[$].[Na+][]}

References:

1. H. M. Klukovich, T. B. Kouznetsova, Z. S. Kean, J. M. Lenhardt and S. L. Craig, *Nat. Chem.*, 2013, **5**, 110-114.
2. J. Wang, T. B. Kouznetsova, Z. Niu, M. T. Ong, H. M. Klukovich, A. L. Rheingold, T. J. Martinez and S. L. Craig, *Nat. Chem.*, 2015, **7**, 323-327.
3. Z. Wang, X. Zheng, T. Ouchi, T. B. Kouznetsova, H. K. Beech, S. Av-Ron, T. Matsuda, B. H. Bowser, S. Wang, J. A. Johnson, J. A. Kalow, B. D. Olsen, J. P. Gong, M. Rubinstein and S. L. Craig, *Science*, 2021, **374**, 193-196.
4. B. H. Bowser, C.-H. Ho and S. L. Craig, *Macromolecules*, 2019, **52**, 9032-9038.
5. J. J. Lessard, G. M. Scheutz, S. H. Sung, K. A. Lantz, T. H. Epps, III and B. S. Sumerlin, *Journal of the American Chemical Society*, 2020, **142**, 283-289.
6. M.-A. B. Kruft and L. H. Koole, *Macromolecules*, 1996, **29**, 5513-5519.
7. I. M. Klein, C. C. Husic, D. P. Kovács, N. J. Choquette and M. J. Robb, *J. Am. Chem. Soc.*, 2020, **142**, 16364-16381.
8. T. Ouchi, B. H. Bowser, T. B. Kouznetsova, X. Zheng and S. L. Craig, *Mater. Horiz.*, 2023, **10**, 585-593.
9. R. S. Rivlin and A. G. Thomas, *J. Polym. Sci.*, 1953, **10**, 291-318.
10. G. S. Grest and K. Kremer, Molecular Dynamics Simulation for Polymers in the Presence of a Heat Bath. *Phys Rev A (Coll Park)* **1986**, *33* (5), 3628–3631. https://doi.org/10.1103/PhysRevA.33.3628.
11. K. Kremer and G. S. Grest, Dynamics of Entangled Linear Polymer Melts: A Molecular-Dynamics Simulation. *J Chem Phys* **1990**, *92* (8), 5057–5086. https://doi.org/10.1063/1.458541.
12. J. K. Johnson, J. A. Zollweg, and K. E. Gubbins, The Lennard-Jones Equation of State Revisited. *Mol Phys* **1993**, *78* (3), 591–618. https://doi.org/10.1080/00268979300100411.
13. D. Frenkel, D. and B. Smit, *Understanding Molecular Simulation: From Algorithms to Applications*; Academic Press, 2002.
14. P. M. Morse, Diatomic Molecules According to the Wave Mechanics. II. Vibrational Levels. *Physical Review* **1929**, *34* (1), 57–64. https://doi.org/10.1103/PhysRev.34.57.
15. S. Plimpton, Fast Parallel Algorithms for Short-Range Molecular Dynamics. *J Comput Phys* **1995**, *117* (1), 1–19. https://doi.org/10.1006/jcph.1995.1039.
