# Supplementary material for: Tuning the Ultimate Strain of Single and Double Network Gels Through Reactive Strand Extension
Source: ACS Cent Sci. 2025 Aug 15;11(10):1882–91. doi: 10.1021/acscentsci.5c00932 (PMC12550619; doi:10.1021/acscentsci.5c00932)
Supplement: Supplementary file 5 [file oc5c00932_si_005.pdf]

oc-2025-009329.R1

Name: Peer Review Information for "Tuning the Ultimate Strain of Single and Double Network Gels Through Reactive Strand Extension"

First Round of Reviewer Comments

Reviewer: 1

Comments to the Author

Molecule-to-material correlation represents a challenge in material design. This work is an excellent example of establishing such a correlation in mechanophore containing networks. Building on their recent success with the cyclobutene copolymers, the authors evaluated how the size of released contour length affects gel toughness in SN and DN topologies, and indeed found a direct correlation. The experiments are well-designed and logical. The aims/questions this work is trying to address are well articulated and the results are well rationalized. This solid work is significant and broadly appealing as it advances our understanding on how small changes in molecular features affect the macroscopic mechanical properties of polymer networks. I recommend publication with a few comments:

1. The difference in toughness between N5 vs N12 (networks containing 5 or 12 carbon side chain loops) is clear. I'm actually surprised the authors observed such differences (to be clear, I'm not questioning their experiments), considering presumably only a very small and uncertain fraction of the mechanophores activate within the networks. Without seeing their data, I would have thought such a small degree of strand extension at molecular scale would not reflect in macroscopic properties. Can the authors offer their insight or some theoretical framework that would support the observation?

2. It's actually rather surprising to me that the control molecule seems to impact the fabrication of the double network (brittleness) as compared to the non-scissile

mechanophores, given the similarities of the as-characterized single network materials (yes it fails earlier, but not that much earlier than SN5...)

3. The SN gels were evaluated with the compression tests. Were they too weak for tensile tests? Or tensile tests not reveal the difference? some comment on tensile evaluation would be helpful to readers.

4. Page 7, bottom, the wording is a little confusing: it is saying that the authors are working with compressible networks, but they are back-calculating parameters which apply to incompressible networks?

5. Page 10, the table has fracture energy Gf but the caption has tearing energy T.

6. Page 11, "the first network in DN5 break at a lower critical strain than in DN12, as observed in the corresponding single networks…In contrast, the fracture of the first network SN12 in DN12 initiates at higher strain, at which point a greater fraction of the stress is transferred to the second network". From Fig. 5 it looks like the yielding behavior in DN12 begins earlier than the complete material failure of DN5, and that the curves are relatively well aligned with each other until the onset of the necking in DN12. I don't quite understand the authors' argument. some clarification is helpful.

7. Why was it chosen to swap mechanophore for AA, instead of simply adjusting the mechanophore:NaAMPS ratio?

Wording:

The authors describe the reaction as cycloelimination. Should it be cycloreversion?

I suggest the authors provide the product structure for readers who not familiar with the mechanochemistry.

Reviewer: 2

Comments to the Author

Zheng, et al. have submitted a manuscript where they explore polymer networks where the strands can extend upon mechanical stimuli. This work seems to follow on a previous paper in Science in 2021 (Reference 14) by a similar group of authors, and the relationship with this previous work seems appropriate. This current work is strong and well-written, and should be published. A few suggestions are given below, mostly for clarification.

1. Is it possible to more clearly annotate and explain the SMFS data shown in Figure 2c? Readers unfamiliar with SMFS will find this difficult to interpret. How does this data verify the predictions in Figure 2b?

2. I think I understand the setup shown in Figure 4cd, but more explanation would be helpful. For example, the word "mirror" seems to point to two different locations in Figure 4d. Perhaps a cartoon could clear up some confusion.

3. How are authors assigning the strain at failure in the compression experiments for the single networks? Is it from the stress-strain curves in Figure 4b? This is hard to see. For example, the authors report 72% for SN5, but the curve looks more like 75%. Can videos of the compression test also be included, as these are mentioned in the methods section?

4. A difference of 72% vs. 78% in compressive failure strains for SN5 vs. SN12 does not seem like much. How statistically significant is this? How many tests were performed?

Author's Response to Peer Review Comments:

Review #1.

Comments:
Molecule-to-material correlation represents a challenge in material design. This work is an excellent example of establishing such a correlation in mechanophore containing networks. Building on their recent success with the cyclobutene copolymers, the authors evaluated how the size of released contour length affects gel toughness in SN and DN topologies, and indeed found a direct correlation. The experiments are well-designed and logical. The aims/questions this work is trying to address are well articulated and the results are well rationalized. This solid work is significant and broadly appealing as it

advances our understanding on how small changes in molecular features affect the macroscopic mechanical properties of polymer networks. I recommend publication with a few comments:

1. The difference in toughness between N5 vs N12 (networks containing 5 or 12 carbon side chain loops) is clear. I'm actually surprised the authors observed such differences (to be clear, I'm not questioning their experiments), considering presumably only a very small and uncertain fraction of the mechanophores activate within the networks. Without seeing their data, I would have thought such a small degree of strand extension at molecular scale would not reflect in macroscopic properties. Can the authors offer their insight or some theoretical framework that would support the observation?

Response. We were actually surprised, too, but there is an explanation. We have added the following text to the manuscript:

The effect of RSE is consistent with expectations based on Lake-Thomas theory, which draws a connection between the toughness of a network and the energy stored in individual network strands that are resisting crack propagating at the point where each strand breaks. The longer the strand, the more energy that is stored. In a typical network, the length of the strands that break is generally taken to be the same as the length of strands in the as-formed networks. In the networks explored here, however, **SN_{12}**, **SN_5**, and **SN_{con}** begin with effectively identical strand lengths, but the strands that ultimately break are quite different in length due to the mechanochemical extension of the strands under highest tension, and greater RSE leads to more strand length in which to store energy. Only a very small fraction of strands undergoes RSE and scission, but these are the strands that are resisting crack propagation. As a result, the capacity for elastic energy to be stored at the front of the propagating crack increases, leading to higher tearing energies.

2. It's actually rather surprising to me that the control molecule seems to impact the fabrication of the double network (brittleness) as compared to the non-scissile mechanophores, given the similarities of the as-characterized single network materials (yes it fails earlier, but not that much earlier than SN5...)

Response. We obviously wish this weren't the case, but the data are the data. A significant contribution is undoubtedly that the degree of swelling changes in water, presumably making all of the networks more brittle. The fact that it is at a point that creates the logistical challenge in making the control DN is, we believe, a coincidence and not a general rule.

3. The SN gels were evaluated with the compression tests. Were they too weak for tensile tests? Or tensile tests not reveal the difference? some comment on tensile evaluation would be helpful to readers.

Response. Agreed. The following text is now in the manuscript:

The brittleness of the SN gels made tensile tests impractical (they break frequently when clamping),...

4. Page 7, bottom, the wording is a little confusing: it is saying that the authors are working with compressible networks, but they are back-calculating parameters which apply to incompressible networks?

Response. The language is confusing. The gels are “compressible” in the sense that they can be compressed in one dimension. They are “incompressible” in the mechanics sense that when deformed the total volume does not change. We have clarified as follows:

For an incompressible network (i.e., volume is constant under deformation),...

5. Page 10, the table has fracture energy Gf but the caption has tearing energy T.

Response. Thank you for catching this! We have corrected the caption.

6. Page 11, "the first network in DN5 break at a lower critical strain than in DN12, as observed in the corresponding single networks...In contrast, the fracture of the first network SN12 in DN12 initiates at higher strain, at which point a greater fraction of the stress is transferred to the second network". From Fig. 5 it looks like the yielding behavior in DN12 begins earlier than the complete material failure of DN5, and that the curves are relatively well aligned with each other until the onset of the necking in DN12. I don't quite understand the authors' argument. some clarification is helpful.

Response. We agree that this argument would benefit from clarification, and we have added the following text to the manuscript:

These observations suggest the following picture. **DN$_5$** and **DN$_{12}$** behave identically until they reach the critical strain where cycloelimination begins. At this point, the first network in both gels softens as hidden length is released. This initial softening effect is expected to be comparatively weaker in **DN$_5$** than in **DN$_{12}$**, as a result of the smaller hidden length in the mechanophores of **DN$_5$**. Consequently, **DN$_5$** demonstrates higher stress resistance against further increases in strain compared to **DN$_{12}$** (Figure 5a). This, in turn, leads to more extensive cycloelimination and bond breaking within **DN$_5$**. This explains the larger

hysteresis (Figure 5c) and greater fractional loss of Young's modulus (Figure 5d) observed for **DN_5**.

Furthermore, the softening of the first network—induced by the release of hidden length—effectively enhances the load-bearing capacity of the second network. It appears that the more significant lengthening in **DN_{12}** results in a relatively stronger second network, which contributes to the overall DN gel's greater extensibility.

7. Why was it chosen to swap mechanophore for AA, instead of simply adjusting the mechanophore:NaAMPS ratio?

Response. We have added the following explanation to the main text of the manuscript:

Acrylic acid was chosen for the replacement instead of NaAMPS in the hope that preserving the counterion might allow a direct comparison of networks with different RSE co-monomer content, although this turned out not to be the case.

Wording:
The authors describe the reaction as cycloelimination. Should it be cycloreversion?

Response. Corrected; thank you.

I suggest the authors provide the product structure for readers who not familiar with the mechanochemistry.

Response. Done, and we have inserted text to guide the reader as follows:

The molecular design is shown in Figure 2, and the reactants and products of the mechanochemical reaction are shown in Figure 2b.

Additional Questions:
Quality of experimental data, technical rigor: Top 1%

Significance to chemistry researchers in this and related fields: Top 1%

Broad interest to other researchers: Top 10%

Novelty: Top 10%

Is this research study suitable for media coverage or a First Reactions (a News & Views piece in the journal)?: Yes

Review #2.

Recommendation: Publish in ACS Central Science after minor revisions noted.

Comments:
Zheng, et al. have submitted a manuscript where they explore polymer networks where the strands can extend upon mechanical stimuli. This work seems to follow on a previous paper in Science in 2021 (Reference 14) by a similar group of authors, and the relationship with this previous work seems appropriate. This current work is strong and well-written, and should be published. A few suggestions are given below, mostly for clarification.

1. Is it possible to more clearly annotate and explain the SMFS data shown in Figure 2c? Readers unfamiliar with SMFS will find this difficult to interpret. How does this data verify the predictions in Figure 2b?

Response. We are grateful for the suggestion, and we have modified the main text as follows:

For both $\mathbf{P_5}$ and $\mathbf{P_{12}}$, the force-separation curves show a reproducible release of the stored length that manifests as a plateau in the force-extension curve. The width of the plateau is observed to extend up to ~44% and ~85% of the initial polymer contour lengths of $\mathbf{P_5}$ and $\mathbf{P_{12}}$, respectively, across a force range of 1500-2000 pN. The transition force observed here is similar to a previously reported RSE mechanophore based on a similarly fused cyclobutane,[14,18] and the shape of the curves are very similar to computational simulations (Figure S9). The observed extensions in the SMFS are close to but less than the maximum theoretical extensions based on molecular models that show that each cycloreversion in $\mathbf{P_5}$ extends its contour length by 5.9 Å, whereas the extension per event in $\mathbf{P_{12}}$ is 15.2 Å (Figure 2b). When these extensions per mechanophore are combined with the mechanophore content determined by NMR spectroscopy, the expected extension of $\mathbf{P_5}$ and $\mathbf{P_{12}}$ are estimated to be 58% and 150%, respectively (Figure S6).

2. I think I understand the setup shown in Figure 4cd, but more explanation would be helpful. For example, the word "mirror" seems to point to two different locations in Figure 4d. Perhaps a cartoon could clear up some confusion.

Response. We have revised and combined Figure 4c and d to improve the clarity of the set up (now Fig. 4c):

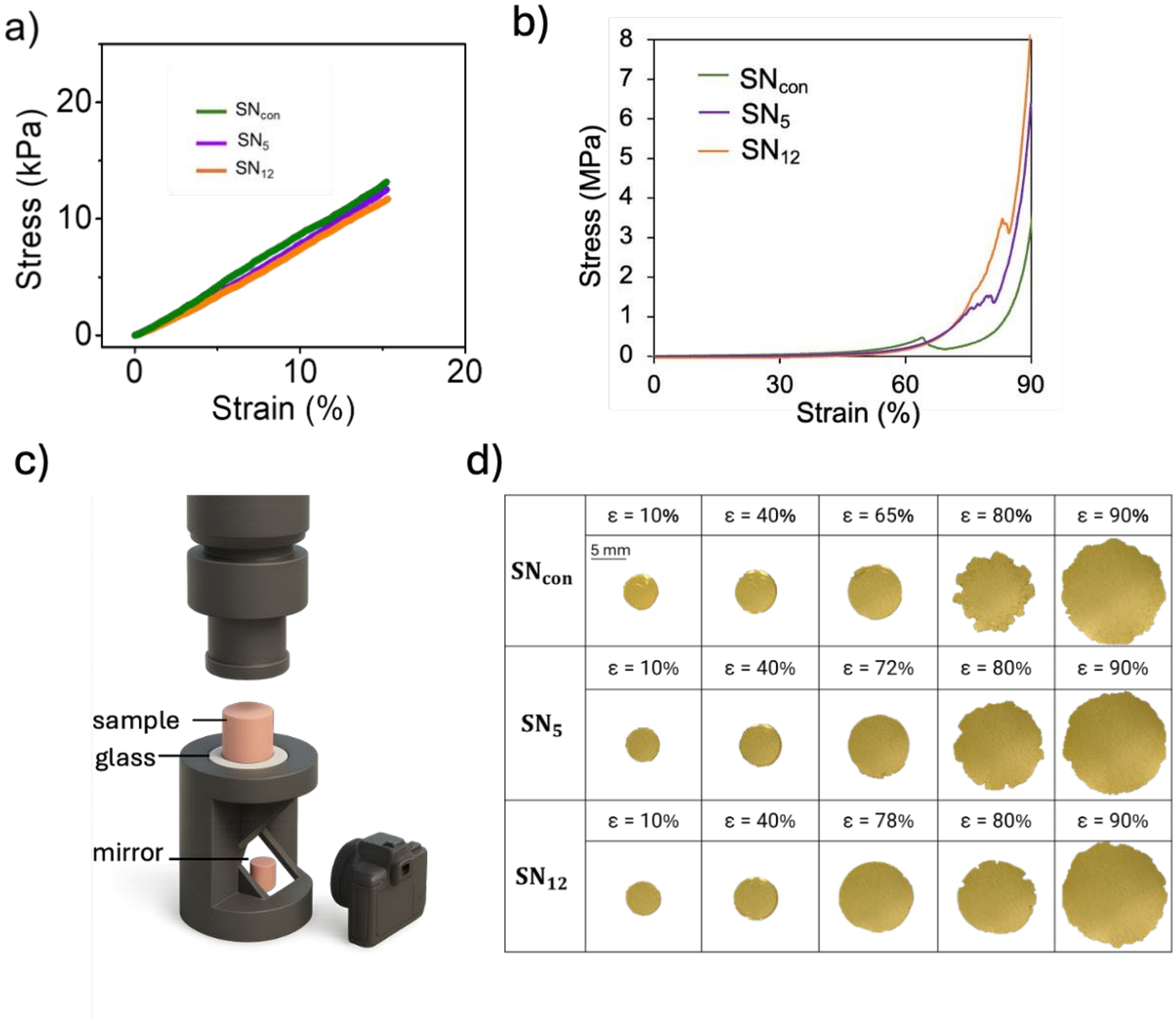

3. How are authors assigning the strain at failure in the compression experiments for the single networks? Is it from the stress-strain curves in Figure 4b? This is hard to see. For example, the authors report 72% for SN5, but the curve looks more like 75%. Can videos of the compression test also be included, as these are mentioned in the methods section?

Response. This is a good catch by the reviewer. Figure 4b accidentally used data from an original round of mechanical testing that was performed on a tester without the optical setup. We have updated the table, added the videos to the SI as requested, and modified the main text as follows:

During compression, the behavior of the gels was recorded using a custom-built setup. This setup enables observation of the sample behavior through a side-mounted camera and mirror system (Figure 4c). The onset of cracking was observed visually before the

significant change in stress strain curve. Therefore, we used the timepoint corresponding to initial crack formation to assign the failure strain, rather than relying solely on force measurements. This approach was applied consistently across all samples to ensure comparability of strain measurements. Representative compression videos are included in the Supporting Information.

4. A difference of 72% vs. 78% in compressive failure strains for SN5 vs. SN12 does not seem like much. How statistically significant is this? How many tests were performed?

Response. The data is reproducible within ± 2% at n = 3 (and the same trend in relative values with only slightly different absolute values was recorded in independent measurements on a different set of gels, but without the optical setup). To clarify, we have added following text to the manuscript.

We conducted compression tests on n = 3 independent replicates for each network formulation (**SN$_{con}$**, **SN$_5$**, and **SN$_{12}$**). The corresponding stress–strain curves are shown in Figure 4b, with three individual curves plotted for each sample.

and

Failure strain was determined as the point at which visible cracking first occurred, identified in real time using the side-mounted camera and mirror system (Figure 4c). Cracking consistently appeared at a compressive strain of 63 ± 2% in **SN$_{con}$**, whereas **SN$_5$** and **SN$_{12}$** incorporating RSE mechanophores consistently break at higher strains of approximately 72 ± 2% and 78 ± 2%, respectively. Thus, the trends in strain at break were reproducible across the mechanical tests and the video evidence, revealing delayed crack initiation in **SN$_{12}$** compared to **SN$_5$**. This higher failure strain in **SN$_{12}$** correlates with its increased molecular extensibility, as evidenced by the SMFS measurements.
